# Cell Morphogenesis Proteins Are Translationally Controlled through UTRs by the Ndr/LATS Target Ssd1

**DOI:** 10.1371/journal.pone.0085212

**Published:** 2014-01-21

**Authors:** Antony G. Wanless, Yuan Lin, Eric L. Weiss

**Affiliations:** Department of Molecular Biosciences, Northwestern University, Evanston, Illinois, United States of America; University of Tokyo, Japan

## Abstract

Eukaryotic cells control their growth and morphogenesis to maintain integrity and viability. Free-living cells are further challenged by their direct interaction with the environment and in many cases maintain a resilient cell wall to stay alive under widely varying conditions. For these organisms, stringent and highly localized control of the cell wall's remodeling and expansion is crucial for cell growth and reproduction. In the budding yeast *Saccharomyces cerevisiae* the RNA binding protein Ssd1 helps control cell wall remodeling by repressing translation of proteins involved in wall expansion. Ssd1 is itself negatively regulated by the highly conserved Ndr/LATS protein kinase Cbk1. We sought to identify mRNA regions that confer Ssd1-mediated translational control. After validating a GFP reporter system as a readout of Ssd1 activity we found that 3′ untranslated regions of the known Ssd1 targets *CTS1, SIM1* and *UTH1* are sufficient for Cbk1-regulated translational control. The 5′ untranslated region of *UTH1* also facilitated Ssd1-mediated translational control in a heterologous context. The *CTS1* and *SIM1* 3′ untranslated regions confer Ssd1 binding, and the *SIM1* 3′ untranslated region improves Ssd1 immunoprecipitation of the endogenous *SIM1* transcript. However, *SIM1*'s 3′ untranslated region is not essential for Ssd1-regulated control of the message's translation. We propose that Ssd1 regulates translation of its target message primarily through UTRs and the *SIM1* message through multiple potential points of interaction, permitting fine translational control in various contexts.

## Introduction

Many single-celled organisms maintain a cell wall. This barrier is crucial for separating the intercellular space from the environment, but presents a problem: it must be continually remodeled for growth to occur [1]–[3]. This is a dynamic process that requires both deposition of new wall material and removal or rearrangement of existing linkages. In fungi, the integrity of the cell wall is crucial for survival, and any action to remodel it is tightly controlled such that polarized growth and proliferation is kept in balance with stress resistance and osmotic stability. In the budding yeast *S. cerevisiae*, cell wall biogenesis and remodeling involves a combination of local wall polymer synthesis and tightly controlled secretion of hydrolases that open up the lattice and allow it to expand. While key components of the budding yeast wall organization system have been discovered [1]–[3], it remains unclear how the opposing extracellular processes of wall synthesis and hydrolysis are kept in balance, properly localized, and coordinated with growth status.

Proper control of wall hydrolases, which could reduce cell integrity if hyperactive, is probably especially crucial for normal cell growth and stress resistance. In budding yeast the mRNA binding protein Ssd1 provides an important part of this control. Ssd1 is highly conserved in fungi; it contains a C-terminal RNaseII – related domain that lacks residues necessary for catalytic function and an N-terminal region with conserved sequence blocks of unknown function and a propensity for prion formation. While Ssd1 can bind bulk RNA [4], large scale analyses of mRNA binding indicate that the protein associates with specific transcripts [5]–[6]. These include mRNAs that encode cell wall related proteins, notably the cell separation chitinase Cts1 and the “SUN” family wall hydrolases Sun4, Sim1, and Uth1. Ssd1 suppresses translation of these wall remodeling proteins, and this activity is important under commonly occurring stressful conditions, such as ethanol-containing growth medium [7]–[8]. Consistent with a role in translational control, Ssd1 is recruited to cytoplasmic “P bodies”, which are discrete cytoplasmic foci at which mRNAs removed from active translation accumulate, and also interacts with proteins involved in the control of translation [6], [9]–[10].

Ssd1's ability to block translation of bound mRNAs is efficiently negatively regulated by the Ndr/LATS family protein kinase Cbk1, which directly phosphorylates Ssd1's N-terminal region [6]. This phosphorylation does not affect Ssd1's ability to bind mRNA, but rather appears to reduce its ability to block the translation of associated messages [6]. Cbk1 is an essential component of a highly conserved regulatory system called the “RAM network” that controls final separation of mother and daughter cells and sustained polarized growth that occurs during mating and bud morphogenesis [11]–[18]. When the function of any component of the RAM network is lost Ssd1 is hyperactivated, causing constitutive translational repression of cell wall remodeling proteins. This loss of hydrolase expression is lethal because it effectively blocks cell wall expansion, severely restricting bud growth and blocking cell proliferation [6]. Similarly, changing the amino acids in Ssd1 that are phosphorylated by Cbk1 to non-phosphorylatable residues creates a highly toxic *ssd1* gain-of-function allele (ssd1-8A) that gives a substantially similar suppression of wall expansion when expressed.

Intriguingly, while Ssd1 clearly modulates translation of some of the mRNAs it binds, the protein has additional functions. While the effect may be indirect, the decay rates of diverse mRNAs is faster in cells that express functional Ssd1, regardless of whether or not these messages associate with Ssd1 [6], and many genes are differentially expressed depending on the presence of functional Ssd1 [19]. A number of Ssd1-associated transcripts are asymmetrically localized in proliferating cells [20]–[21], and Ssd1 has been implicated in subcellular localization of one of these (*SRL1*) [9]. At least one Ssd1-associated message, *CLN2*, is stabilized by Ssd1 binding to its 5′ untranslated region [22]. It is not known if Cbk1 regulates Ssd1-mediated stabilization of *CLN2* or if this 5′UTR-mediated association is related to Ssd1's function as a translational repressor.

Ssd1's apparently diverse functions may reflect the underlying complexity of messenger RNA particle (mRNP) organization, in which different complements of associated regulatory proteins confer distinct mRNA behavior. It is unclear how Ssd1 associates with mRNAs, and understanding this could illuminate what dictates the composition of Ssd1-containing mRNPs. The motif A[G/U]UCAUUCCUU is significantly enriched in 5′ untranslated regions of mRNAs that associate with Ssd1 in affinity purification experiments [5], and a portion of the *CLN2* 5′UTR containing a sequence matching this motif mediates Ssd1 association [22]. For brevity, we refer to this motif as the “SEE” (**S**sd1 **E**nriched **E**lement). While it occurs with elevated frequency in Ssd1-associated mRNAs, the SEE is not present in all of them, and the motif has not been directly shown to be sufficient for Ssd1-mRNA binding or Ssd1-regulated translational control. Ssd1 also interacts with the poly-A binding protein Pab1 [23], suggesting that Ssd1-mRNP interactions are complex. We sought to determine if the 5′UTR of an Ssd1-regulated message is sufficient to confer translational regulation on an otherwise unregulated transcript and if Ssd1 acts through other regions of target messages. Given the extensive role of 3′UTR sequences in post-transcriptional regulation [24]–[27], our study encompasses both 5′ and 3′UTRs. Here, we expand analysis of Cbk1-regulated translational control through Ssd1 [6] by identifying *cis*-acting regions of Ssd1-bound transcripts that are sufficient for translational control. We show that the 3′UTRs of a subset of Ssd1 target transcripts mediate translational repression, and that in at least one case a 5′UTR is sufficient for such regulation. We propose that this UTR-encoded system allows Ssd1 to exert coordinated control over expression of proteins influencing morphogenesis and cell resilience.

## Materials and Methods

### Yeast strains and plasmids

We constructed all yeast in the S288c background strain BY4741 (Open Biosystems). We constructed GFP reporter plasmids the Drag and Drop recombinant cloning method [28], using known annotations of 5′ and 3′ UTR boundaries [29]–[30] to generate primers for UTR integration into pGREG vectors. We replaced the pGREG *GAL1,10* promoter with the *TEF1* or *ADH1* promoters and their 5′UTRs from the PCR Toolbox vectors pYM-N18 or pYM-N6 [31], respectively, by subcloning at SacI-SpeI. We made destabilized GFP^PEST^ reporters by PCR-mediated stitching of the 534 nucleotides encoding the 178 C-terminal residues of Cln2 [32], followed by a stop codon, to the 3′UTR of interest and subsequent recombinant cloning into a pGREG576 N-terminal GFP vector. We sequenced all plasmids and checked fusion protein expression by western blotting against GFP (Roche cat. no. 11814460001). We replaced the endogenous 3′UTR of *SIM1* using homologous recombination to integrate a PCR product encoding the *CYC1* 3′UTR at the 3′ end of *SIM1*, following the stop codon.

### Flow cytometry analysis of GFP reporters

We grew samples for flow cytometry to mid-log (OD_600_≈0.6) in YP (yeast peptone) rich media and washed into PBS, or in YNB (yeast nitrogen base) synthetic media for direct analysis. We grew GFP^PEST^ time course samples to OD_600_≈0.4 and treated cells with 25 µM 1NA-PP1 or an equivalent volume of DMSO vehicle. We removed 1 mL from each sample every hour following treatment, fixed these time points in ice-cold ethanol and stored them in the dark for cumulative analysis. We used a Becton Dickinson LSR II flow cytometer with a 488 nm excitation laser and a 530 nm emission filter (FITC), kept laser power settings the same between experiments and collected a minimum of 10,000 cells per sample. We analyzed flow cytometric data using FACSDiva (Becton Dickinson) or FlowJo (Tree Star) software. Briefly, we gated cells from debris by forward-scatter/side-scatter plots and set baseline GFP fluorescence gates using side-scatter/FITC plots of cells expressing no GFP reporter. We adjusted experiments showing deviations in cell size between samples by taking the ratio of mean FITC fluorescence to mean forward-scatter signal; this is indicated as FSC-normalized. We used mean fluorescence intensity (MFI) statistics of both the population above baseline FITC autofluorescence and MFI of the entire population to make comparisons between reporters. We analyzed mRNA collected from the same flow cytometry samples by real-time qPCR (see below) to evaluate translation efficiency as noted.

### Polysome profiling of GFP reporters and Northern blot analysis

We did polysome profiling and subsequent Northern blotting analysis of fractionated RNA as previously described [6]. We amplified probes from genomic DNA using the following primers and labeled with Ready to Go beads (Amersham) and α32P-dCTP: *GFP* 5′ GTGAAGGTGATGCAACATAC and 5′ TGGTTGTCTGGTAAAAGGAC, *PGK1* 5′ GAATTGTTGCTGCTTTGCCA and 5′ TTCTCCAAAGCCTTACCGAA. We performed AUC (Area Under the Curve) analysis of relative polysome and monosome association across three replicate experiments using Prism (GraphPad).

### Purification of Ssd1-associated mRNA

We purified Ssd1-associated transcripts in cells expressing Ssd1-TAP from the endogenous *SSD1* locus and in cells expressing untagged Ssd1 as previously-described [33], but scaled-down 10-fold. We modified the antibody-mediated RNA immunoprecipitation by Ssd1-TAP as follows: we incubated lysates with 2 µg anti-TAP rabbit polyclonal antibody (Thermo-Fisher Pierce cat. no. CAB1001) for 30 minutes at 4°C, followed by 2 hour incubation at 4°C with recombinant Protein G-sepharose 4B beads (Life Technologies). We recovered mRNA from Protein G-sepharose 4B beads (washed as described in [33]) using the MasterPure Yeast RNA Purification Kit (Epicentre Technologies). We determined relative abundance of recovered mRNA by real-time qPCR. We reverse transcribed (Promega) 2.5 µg RNA primed only with oligo dT and detected messages by incorporation of SYBR green (Life Technologies) into amplicons generated with the following primers: *SUN4* 5′ AACTTTGGCGCTGGTTCTTC and 5′ TCATCAGCGGCGACAATTTT, *GFP* 5′ TGGAAGCGTTCAACTAGCAG and 5′ AAAGGGCAGATTGTGTGGAC, *SIM1* 5′ TCTGGTGCCATCGTGTCTGCTTTA and 5′ AAACGTATTTGTACGCAACGGCCC, *ACT1* 5′GGTTATTGATAACGGTTCTGTATG and 5′ ATGATACCTTGGTGTCTTGGTCTAC. We obtained relative concentrations using efficiency-corrected standard curves generated from 10-fold serial dilutions of intact *S. cerevisiae* genomic DNA.

### Assay of secreted target proteins

We evaluated abundance of Ssd1 target proteins by assaying newly replenished yeast growth media. We treated logarithmically growing cells with 10 µM 1NA-PP1 or an equivalent volume DMSO vehicle for 1 hour. At OD_600_≈0.5 we washed cells twice into new YPD rich media containing 10 µM 1NA-PP1 or an equivalent volume DMSO and continued growth at room temperature. We removed 1 mL aliquots of cells in growth media at 5, 15, and 30 minutes post-media replenishment and treated these samples with sodium azide at a final concentration of 20 mM to stop growth. We pelleted the cells and treated 800 µL supernatant media with 89 µL ice cold 100% trichloroacetic acid (TCA), incubating on ice for 20 minutes to precipitate secreted proteins. We spun TCA-precipitated media at top speed in a 4°C microcentrifuge, aspirated the media following centrifugation, and washed the resulting precipitated protein pellet in acetone. We air-dried precipitated protein pellets and resuspended them in Tris pH 9.4-buffered SDS-PAGE loading buffer. We lysed cell pellets from each time point to assay internal protein content by alkaline disruption [34].

### Yeast 3-hybrid assay for Ssd1-mRNA interactions

The yeast 3-hybrid system used in this study was provided by Dr. Marvin Wickens (University of Wisconsin), and includes a yeast strain YBZ-1 [35] which encodes a LexA-MS2 coat protein fusion and encodes *HIS3* under control of the *lexA* operator. We amplified the *CTS1* 3′UTR, *CYC1* 3′UTR or *CLN2* 5′UTR using specific oligonucleotides encoding SmaI restriction endonuclease sites at the ends of the amplicon. We cloned these UTR-encoding products into pIII/MS2-1 or pIII/MS2-2 at SmaI, resulting in hybrid RNAs where the test UTR is downstream or upstream of the MS2 loop sequences, respectively. We amplified Ssd1(1-862)-8E from the ELY1294 yeast strain [6] using oligos encoding a BamHI restriction site at the 5′ end and a XhoI restriction site at the 3′ end of the amplicon. We ligated this product into pACT2 at BamHI-XhoI in frame with the Gal4-AD. We cotransformed hybrid RNA and AD-Ssd1(1-862)-8E plasmids into YBZ-1 and maintained them on YNB media lacking uracil and leucine. We grew all strains to stationary phase, diluted to a final OD_600_ of 0.1, and spotted in 5-fold serial dilutions on YNB media lacking: uracil and leucine; uracil, leucine and histidine; or uracil, leucine and histidine supplemented with 1 mM 3-aminotriazole. We incubated plates at 24°C for 3 days and imaged on a CHEMIGENIUS2 (Syngene) system. We used the kit's included *IRE-MS2* RNA and AD-IRP plasmids as a positive control and cotransformed IRE-MS2 with AD-Ssd1(1-862)-8E as a negative control.

## Results

Gene products responsible for cell wall remodeling in *S. cerevisiae* help balance morphogenesis with stress resistance [1]–[3]. Many transcripts encoding these proteins are associated with Ssd1 [5]–[6], [22], [36]. We have previously shown that phosphoregulated translational control of specific bound transcripts is one of Ssd1's principal functions [6]. The *cis*-elements that confer this mode of RNA binding and translational control has not been described; to this end, we developed a reporter system to evaluate the effect of elements from Ssd1-bound transcripts on GFP expression in a heterologous context. We reasoned that Ssd1 recognition elements are present in untranslated regions (UTRs) of the cohort of Ssd1-bound messages, consistent with existing models of mRNA binding proteins in translational control [5].

We selected GFP as a reporter for translational control because its abundance can be quantitatively evaluated. To create heterologous reporter constructs, we used existing plasmid collections (see Materials and Methods) with different promoters (*P_TEF1_* and *P_ADH1_*), these promoters' associated 5′UTRs, and the *CYC1* 3′UTR. None of the messages bearing the UTRs present in these plasmid systems show significant enrichment in microarray analysis of Ssd1-bound transcripts [6]. Thus, we used these 5′ and 3′UTRs as negative controls in our experiments. To find transcript elements that confer Ssd1-mediated translational repression we used a candidate-based approach by selecting untranslated regions from known mRNA targets of Ssd1 and testing their ability to modulate expression of the GFP reporter system. The boundaries of many UTRs in budding yeast are well-established [29]-[30], allowing us to design reporter constructs using existing data. We asked if 5′UTRs or 3′UTRs from the Ssd1-bound transcripts *CTS1, SUN4* and *UTH1* confer Ssd1 translational regulation of a GFP reporter protein.

### 3′UTRs of Ssd1-target transcripts confer differential expression of GFP reporters

**Figure 1:**
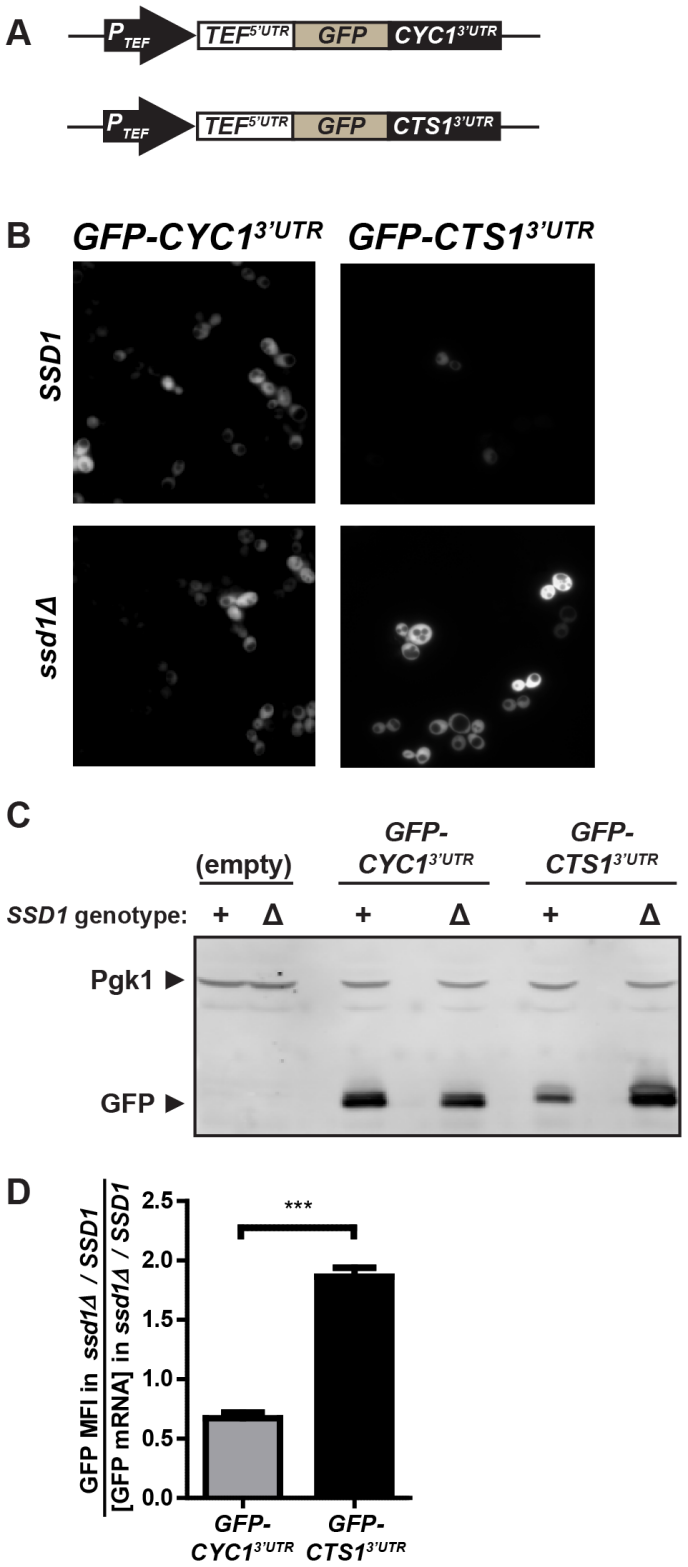
GFP bearing the Ssd1-bound transcript *CTS1's* 3′UTR is differentially expressed depending on Ssd1 genotype. *SSD1* (ELY700) or *ssd1Δ* (ELY853) cells were transformed with [*P_TEF1_-GFP-CYC1^3′UTR^*] or [*P_TEF1_-GFP-CTS1^3′UTR^*], maintained under G418 selection. **(A)** Diagram of exogenous reporters used in this figure. **(B)** Logarithmically-growing cells expressing these reporters in both strain backgrounds were examined by fluorescence microscopy. Representative images of cells expressing [*P_TEF1_-GFP-CYC1^3′UTR^*] showing minimal variance in GFP expression in *SSD1* or *ssd1Δ*, but cells expressed lower levels of [*P_TEF1_-GFP-CTS1^3′UTR^*] in *SSD1* than *ssd1Δ*. All images contrast adjusted in OpenLab software using identical settings. **(C)** Lysates of the same cells used in (B) were analyzed by Western blot against GFP and the housekeeping gene Pgk1, with equal numbers of cells processed for each strain. Western blots confirm lower GFP levels in *SSD1* cells when GFP is expressed with the *CTS1* 3′UTR, while Pgk1 levels are invariant. **(D)** Estimation of translation efficiency of *GFP-CYC1^3′UTR^* and *GFP-CTS1^3′UTR^* reporters, determined through division of GFP MFI (mean fluorescence intensity) by relative GFP transcript abundance (shown in Figure S1A), shows significant repression of [*P_TEF1_-GFP-CTS1^3′UTR^*] in *SSD1* cells. Data in (D) represent three independent trials. Error bars represent ± SEM, *** indicates P-value<0.001, ‘ns’ indicates P-value>0.05 at 95% confidence intervals as calculated by unpaired two-tailed Student's t-test.

We examined the ability of 3′UTRs from Ssd1-bound mRNAs to confer Ssd1-mediated translational regulation. These regions do not contain the SEE (Ssd1 Enriched Element), a motif that occurs with elevated frequency in mRNAs that associate with Ssd1 [5]. The *CTS1* 3′UTR, when present in the heterologous context of a *TEF1* 5′UTR and GFP open reading frame under control of a *TEF1* promoter (Figure 1A) shows markedly better expression in *ssd1Δ* cells than in cells containing *SSD1*. We did not see this *SSD1*-dependent expression difference in a control reporter construct containing the same TEF1 promoter, *TEF1* 5′UTR, GFP open reading frame, and the *CYC1* 3′UTR (Figure 1B). Western blotting against whole cell extracts containing either the test *CTS1* 3′UTR or control *CYC1* 3′UTR reporters confirms an Ssd1-dependent effect on GFP reporter expression; this effect is not observed on the housekeeping protein Pgk1 (Figure 1C). Given the heterogeneity of GFP expression in our reporter-containing populations, we quantified the fluorescence of the GFP-positive population using flow cytometry. Cells expressing the control *CYC1* 3′UTR reporter showed little difference in the population mean fluorescence intensity (MFI) when Ssd1 was absent (0.87-fold change in expression in *ssd1Δ* compared to *SSD1*), while cells containing the test *CTS1* 3′UTR construct exhibited a nearly three-fold, highly significant increase (p<0.0001) in MFI in *ssd1Δ* cells compared to *SSD1* cells (Figure S1A). Mean Fluorescent Intensity of the putative Ssd1-regulated *CTS1* 3′UTR reporter is roughly half that of the control *CYC1* 3′UTR reporter in *SSD1* cells, whereas in *ssd1Δ* cells, the *CTS1* 3′UTR reporter is expressed at roughly 1.5-fold the level of the control reporter (Figure S1B). This observed difference in expression similar to that observed for other 3′UTR reporters of translational repression [37] and is highly significant (p<0.0001).

When *SSD1* is deleted the steady-state levels of many mRNAs increase, whether or not they associate with Ssd1 [6]. To determine if the transcript abundance of both control and hypothetically Ssd1-regulated GFP constructs respond similarly to deletion of *SSD1* we measured GFP message levels in cells grown in the cultures used for flow cytometric analysis. We found that abundance of GFP construct mRNAs with either the control *CYC1* 3′UTR or the Ssd1-regulated *CTS1* 3′UTR were modestly elevated in *ssd1Δ* cells compared to *SSD1* cells, with no statistically significant difference (p = 0.4764) (Figure S1C). This is consistent with prior demonstration that Ssd1 depresses many transcript levels and does so indiscriminately. Importantly, the small variance in mRNA levels expressed from our GFP reporters indicates that changes in fluorescence from the Ssd1-regulated reporter (Figures S1A and S1B) reflect differences in translation levels. We calculated translation efficiency of our GFP reporters in *ssd1Δ* and *SSD1* cells by dividing the fold change in MFI by the fold change in transcript abundance, and found that after accounting for changes in mRNA abundance due to the absence of Ssd1, the *CTS1* 3′UTR shows markedly increased translation efficiency when Ssd1 is absent, while the *CYC1* 3′UTR does not (Figure 1D).

### 3′UTR-mediated Ssd1 translational control is promoter-independent

**Figure 2:**
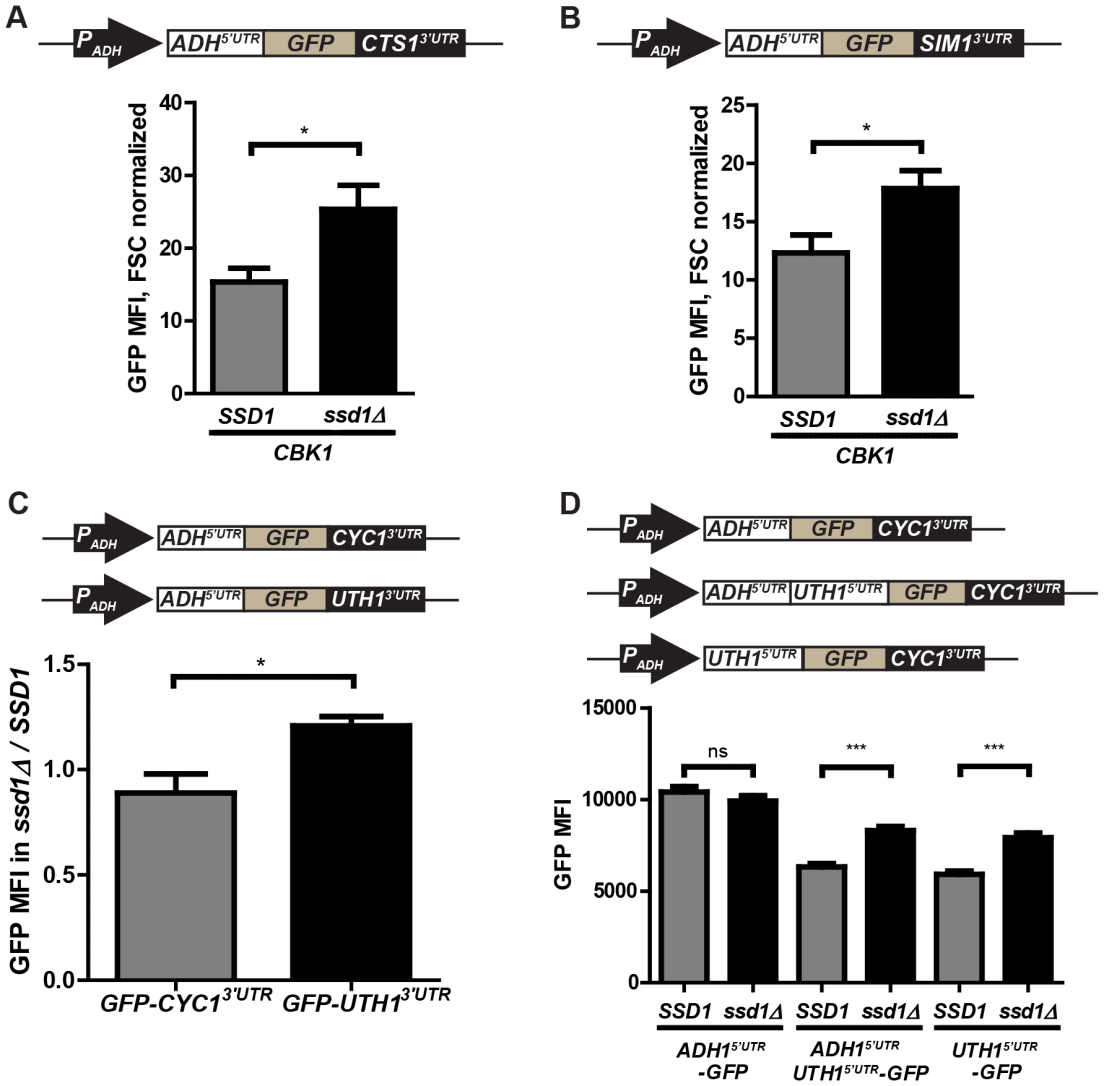
Ssd1 regulates translation of GFP reporters with 3′UTRs from Ssd1-associated transcripts or the *UTH1* 5′UTR. **(A)** *CTS1* 3′UTR reporter GFP MFI difference in *SSD1* and *ssd1Δ* is promoter and 5′UTR independent, as flow cytometry reveals significant Ssd1-dependent differences in expression. **(B)** *SIM1* 3′UTR confers significant Ssd1-dependent expression variances on GFP expressed from the *ADH1* promoter. **(C)** GFP expressed from ADH1 promoter in the context of the *UTH1* 3′UTR is elevated in *ssd1Δ* compared to *SSD1* cells, while the *CYC1* 3′UTR confers no such effect on GFP expression. **(D)** *UTH1* 5′UTR confers decreased expression of GFP in *SSD1* cells, either when present as the only 5′UTR element or expressed in tandem with the *ADH1* 5′UTR, suggesting its effect is context-independent. In (A) and (B), fluorescence data were corrected for variations in cell size apparent in forward scatter (FSC) measurements as described in Materials and Methods. (A) through (D) represent three independent trials. Error bars represent ± SEM, *** indicates P-value<0.001, ** indicates P-value of 0.001 to 0.01, * indicates P-value 0.01 to 0.05, and ‘ns’ indicates P-value>0.05 at 95% confidence intervals as calculated by unpaired two-tailed Student's t-test.

To confirm that the *CTS1* 3′UTR confers context independent Ssd1-regulated translational control we measured expression of GFP from a construct driven by the constitutive *ADH1* promoter and containing the *ADH1* 5′UTR, combined with the *CTS1* 3′UTR. This *P_ADH1_-GFP-CTS1^3′UTR^* reporter showed elevated expression in cells lacking Ssd1 (p = 0.0402), further confirming that the *CTS1* 3′UTR confers Ssd1-mediated translational regulation (Figure 2A). These findings demonstrate that the 3′UTR from the Ssd1-bound transcript *CTS1* can confer Ssd1-dependent changes in expression in two heterologous contexts, and that these changes are likely post-transcriptional, facilitated through Ssd1-mediated translational regulation.

We tested 3′UTRs of several other transcripts that bind Ssd1 and exhibit Ssd1-mediated translational regulation in our GFP reporter system. We found that GFP expressed from an *ADH1* promoter with the *ADH1* 5′UTR and the 3′UTR from the Ssd1-bound transcript *SIM1* showed consistently lower MFI (p = 0.0444) in cells expressing functional Ssd1 (Figure 2B). We saw a similar statistically significant effect using a GFP reporter with the 3′UTR of the Ssd1-bound transcript *UTH1* (p = 0.0126) (Figure 2C). In contrast, GFP reporter constructs with 3′UTRs from the Ssd1-bound transcripts *SUN4, TOS1* and *SCW10* did not show statistically significant changes in GFP expression dependent on the presence of Ssd1 (Figure S2A). Thus, while 3′UTRs of Ssd1-bound messages do not always confer Ssd1-dependent regulation of GFP translation, 3′UTRs of *CTS1, SIM1 and UTH1* are sufficient for this. While statistically significant, differences between reporter expression in *SSD1* and *ssd1Δ* cells are generally modest. As discussed further below, this is consistent with strong negative regulation of Ssd1 by the Ndr/LATS kinase Cbk1 in proliferating cells in which the wall is rapidly growing [6]. Additionally, we propose that relatively subtle coordinated translational control has appreciable effects on cell wall organization because Ssd1 regulates a cohort of messages involved in similar processes.

### The UTH1 5′UTR confers translational control, while CTS1 and SUN4 5′UTRs do not

We determined if 5′UTR elements from Ssd1-bound transcripts confer Ssd1-dependent expression changes in our GFP reporter system. Of the 5′UTRs tested, only the *UTH1* 5′UTR showed a significant Ssd1 mediated effect on GFP expression, while the control reporter with the *ADH1* 5′UTR showed insignificant Ssd1-dependnent change in MFI (p = 0.2237) (Figure 2D). Our GFP reporters for testing the 5′UTR of *UTH1* contained either the *UTH1* 5′UTR alone or a tandem *ADH1-UTH1* 5′UTR in the presence of a GFP open reading frame and a *CYC1* 3′UTR. Both test constructs showed highly significant (p<0.0001) elevation of GFP expression in the absence of Ssd1, indicating that the *UTH1* 5′UTR confers Ssd1-mediated regulation of translation in the presence of additional 5′UTR elements. The 5′UTRs from the Ssd1-bound messages *CTS1* and *SUN4* did not confer a similar effect (Figure S2B). Both *UTH1* and *CTS1* 5′UTRs contain the SEE present in some Ssd1 targets, while the *SUN4* 5′UTR lacks this motif [5]. Thus, these data show that presence of the SEE is not sufficient for translational control. However, these UTRs are not strictly comparable: the *CTS1* 5′UTR contains a single SEE while the *UTH1* 5′UTR contains four.

### 3′UTR reporters are sensitive to Ssd1's phosphorylation state

The Ndr/LATS kinase Cbk1 negatively regulates Ssd1's translational repression of associated mRNAs; this inhibition is probably very efficient in rapidly growing cells [6]. While proteins encoded by Ssd1-bound transcripts are more abundant in *ssd1 Δ* cells, translational repression is considerably stronger when Cbk1's phosphorylation of Ssd1 is compromised [6], and total loss of Cbk1 function is lethal in cells that express functional Ssd1 [4], [11], [38]–[43]. To study the effect of hyperactive Ssd1 on our GFP reporters we used the *cbk1-as* allele, which encodes a mutant Cbk1 kinase that is specifically inhibited by the otherwise innocuous cell permeable compound 1NA-PP1 [44]–[45]. While the kinase encoded by *cbk1-as* is functional in the presence of the vehicle DMSO it is hypomorphic, resulting in reduced kinase activity compared to that of wild-type *CBK1* [14]. Thus, DMSO-treated (uninhibited) *cbk1-as* cells exhibit elevated Ssd1 translational repression, and 1NA-PP1-treated *cbk1-as* exhibit full Ssd1 hyperactivation.

**Figure 3:**
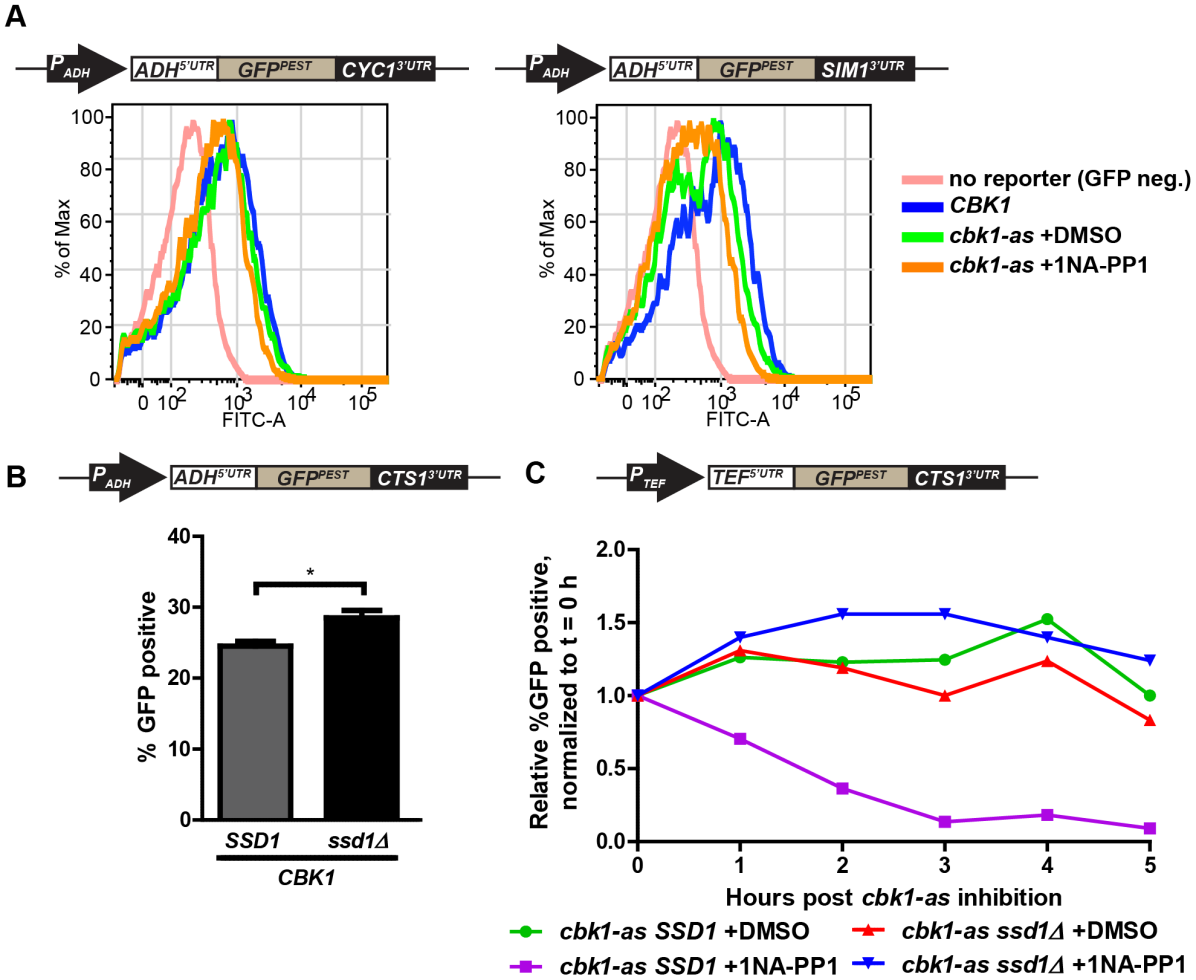
Destabilized GFP reporters show Cbk1-phosphoregulation of Ssd1-dependent changes in expression. **(A)** Destabilized GFP-Cln2^PEST^ bearing the *SIM1* 3′UTR shows moderate shifts in population fluorescence depending on the phosphorylation state of Ssd1, but a destabilized GFP bearing the *CYC1* 3′UTR remains unaffected. Ssd1 phosphorylation state was modulated by the introduction of the hypomorphic *cbk1-as* allele and treatment of these cells with DMSO or 1NA-PP1. **(B)** Destabilized GFP-Cln2^PEST^ harboring the Ssd1-regulated *CTS1* 3′UTR responds to Ssd1 hyperactivation through Cbk1 inhibition. A significant difference in GFP levels was observed by flow cytometry between *cbk1-as SSD1* cells treated with DMSO or 1NA-PP1 through the fraction of the population above baseline fluorescence or. The fraction of cells expressing destabilized GFP was significantly dependent on the presence of Ssd1 (compare *CBK1 SSD1* to *CBK1 ssd1Δ*). **(C)** Prolonged Cbk1 inhibition results in complete depletion of GFP fluorescence in cells expressing an Ssd1-regulated reporter. Flow cytometry was performed on cells fixed at one hour intervals as described in Materials in Methods. We report the relative %GFP positive at each time point t>1 h as a fold change relative to the %GFP positive population at t = 0 h. Additional controls shown in Figure S4C. (A) and (C) are representative trials of replicated experiments. Data in (B) represent three independent trials. Error bars represent ± SEM, ** indicates P-value of 0.001 to 0.01 and * indicates P-value 0.01 to 0.05 at 95% confidence intervals as calculated by unpaired two-tailed Student's t-test.

GFP reporters bearing the *CTS1* 3′UTR or *SIM1* 3′UTR expressed in uninhibited *cbk1-as* cells showed a slight decrease in MFI of the population, with no significant additional reduction in MFI with 1 hour *cbk1-as* inhibition (Figures S3A and S3B). In budding yeast, the half-life of GFP (t½≈7 h) [46] is much longer than the period of a typical cell cycle (∼1.5 h), obscuring changes in translation rate in 1 hour inhibition experiments. To address this, we assessed translational repression using a GFP constructs destabilized by appending the PEST domain from Cln2 to the GFP C-terminus (estimated half-life of 30 minutes) [32]. In cells expressing destabilized GFP^PEST^ in the context of the *ADH1* 5′UTR and the *SIM1* 3′UTR, the maximal fluorescence of the GFP-positive population, visualized in a flow cytometry histogram, was inversely correlated with Ssd1 activity (Figure 3A). With Cbk1 inhibition, the left edge of the histogram population overlays that of the untransformed GFP negative population, showing that *P_ADH1_-GFP^PEST^-SIM1^3′UTR^* expression is repressed in this condition. We did not see a similar change in expression or depletion of signal to background levels when we expressed a control destabilized reporter *P_ADH1_-GFP^PEST^-CYC1^3′UTR^* in the hypomorphic *cbk1-as* background or in cells treated with 1NA-PP1. Destabilized GFP^PEST^ flanked by the *ADH1* 5′UTR and *CTS1* 3′UTR and expressed from a constitutive *ADH1* promoter showed statistically significant reduction in the fraction of the population exceeding the GFP baseline threshold when Ssd1 is present (p = 0.0186) (Figure 3B).

Destabilized GFP^PEST^ expressed from an *ADH1* or *TEF1* promoter bearing the Ssd1-regulated *CTS1* 3′UTR also showed similar reduction in destabilized GFP reporter expression by Western blotting when Ssd1 is present, and that GFP expression is further depleted by expression in the *cbk1-as* background. We found that the *CTS1* 3′UTR Ssd1 reporter behaves similarly irrespective of promoter identity (Figure S4A). Several reports have noted the importance of Ssd1 in stressful conditions such as ethanol-rich growth medium, and offer evidence that Ssd1 regulates cell wall remodeling proteins under these circumstances [7]–[8]. Applying these observations to our GFP reporter system, we grew cells expressing control *CYC1* 3′UTR or test *CTS1* 3′UTR GFP^PEST^ reporters in rich media supplemented with 5% (v/v) ethanol to test if these stress conditions would further stimulate Ssd1 activity. While the cells harboring the *CTS1* 3′UTR reporter in rich media alone showed a modest shift in the population histogram to lower GFP fluorescence in *cbk1-as* than *CBK1* cells, *cbk1-as* cells grown in 5% ethanol showed a dramatic change in the fluorescence profile that is not observed in *CBK1* cells. This effect also depended on the presence of an Ssd1-regulated 3′UTR; in the same strains under the same conditions, the *GFP^PEST^-CYC1^3′UTR^* reporter showed no reduction in fluorescence in the presence of the hypomorphic *cbk1-as* and no response to ethanol treatment in *cbk1-as* cells (Figure S4B). These results confirm that the 3′UTRs from the Ssd1-associated transcripts *SIM1* and *CTS1* confer translational control that is sensitive to Ssd1 activity, and that this does not occur when an unassociated 3′UTR is present.

### Prolonged Cbk1 inhibition results in complete depletion of Ssd1 reporters

While statistically significant, differences in translation of Ssd1-regulated mRNAs between rapidly proliferating *SSD1* and *ssd1 Δ* cells were relatively subtle. In contrast, extended 1NA-PP1 treatment of *cbk1-as* cells should cause persistent hyperactivation of Ssd1 and substantial translational suppression of mRNAs under its translational control, including our GFP reporters. To test this, we grew *cbk1-as* or *cbk1-as ssd1Δ* cells expressing the Ssd1-responsive *P_TEF1_-GFP^PEST^-CTS1^3′UTR^* reporter to mid-log (OD_600_≈0.5) and split the cells into DMSO-treated and 1NA-PP1-treated populations. We allowed treated and untreated cells to grow for 6 hours, fixing samples of each culture at 1 hour intervals for later analysis by flow cytometry. GFP fluorescence was dramatically depleted after 6 hours in *cbk1-as* cells treated with 1NA-PP1, but persisted at levels similar to the beginning of the time course in *cbk1-as* cells treated with DMSO and in *cbk1-as ssd1Δ* cells treated with either 1NA-PP1 or DMSO (Figure 3D). Destabilized GFP reporters were fully depleted only when the *CTS1* 3′UTR was present, as the fluorescence of cells expressing GFP^PEST^ with the *CYC1* 3′UTR control reporter in the same conditions were not depleted even under Ssd1 hyperactivation (Figure S4C). Taken together, these results show that translation of a reporter protein ORF from an mRNA with an Ssd1-regulated 3′UTR is shut off when Ssd1 is no longer inhibited by Cbk1.

### 3′UTR reporters are enriched in monosomes when Ssd1 is hypophosphorylated

**Figure 4:**
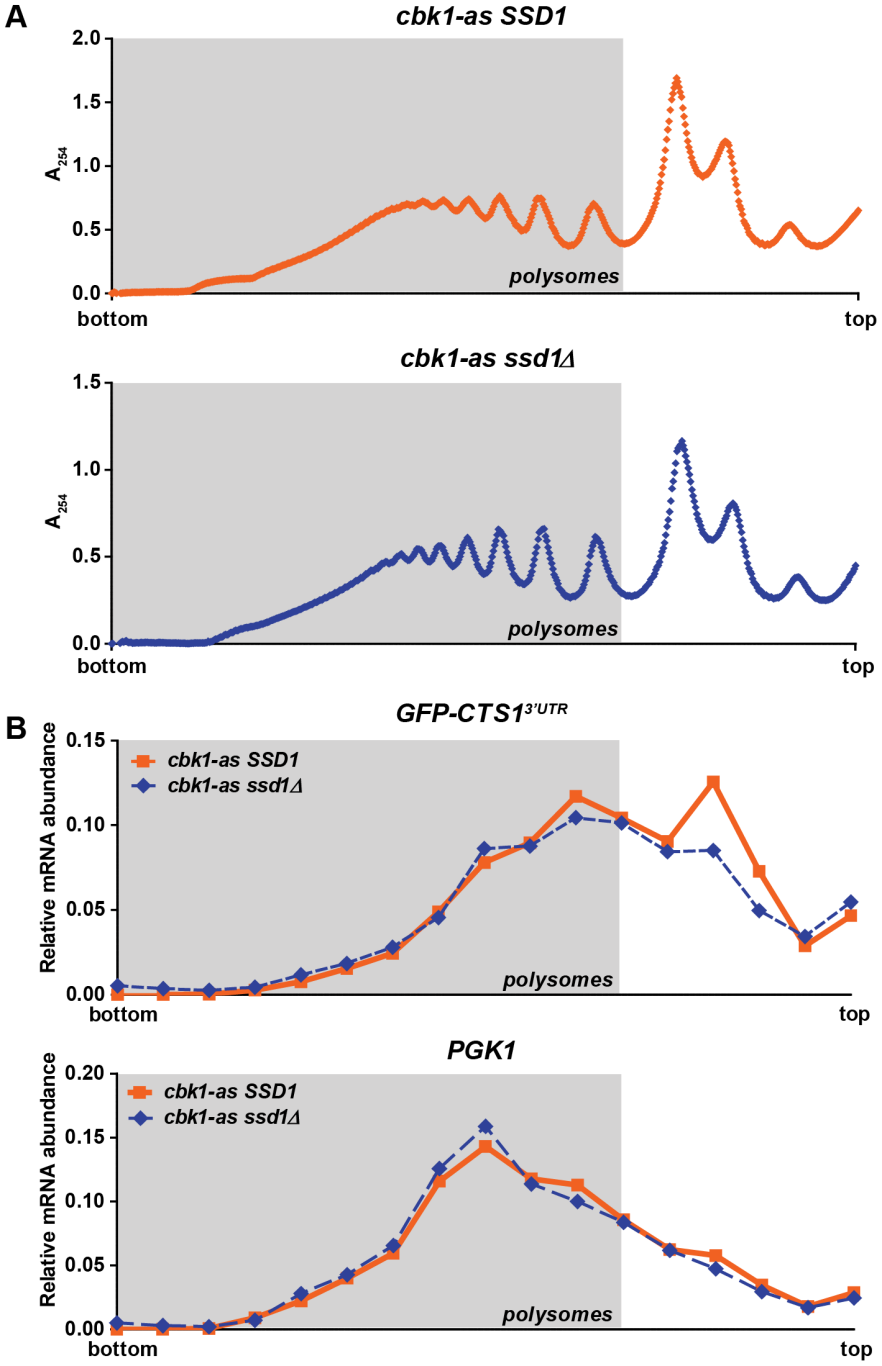
Ssd1-regulated expression of GFP reporter is due to changes in transcript ribosomal occupancy. Polysome profiling of RNA extracts followed by Northern blot analysis of RNA fractions from across the polysome gradient were used to analyze ribosomal occupancy of the *P_TEF1_-GFP-CTS1^3′UTR^* reporter in 1NA-PP1-treated *cbk1-as SSD1* and *cbk1-as ssd1Δ cells*. **(A)** Bulk polysome A_254_ trace of fractionated sucrose gradients reveals fractions containing polysome-associated mRNA (highlighted by gray chart area) and monosome-associated mRNA (towards top of gradient). No changes in bulk translation are observed when Ssd1 is present (top trace) compared to when Ssd1 is absent (bottom trace). **(B)** Quantification from Northern blots against GFP of signal intensity across the polysome gradient reveal that the *GFP-CTS1^3′UTR^* transcript is enriched in monosomes when Ssd1 is present and hyperactivated (compare solid line with square points to dashed line with diamond points). No difference in ribosomal occupancy was observed in Northern blots against the housekeeping gene Pgk1. (A) and (B) are representative plots from three replicate experiments; additional replicates are presented in Figure S5.

Ssd1's control of cell wall protein expression is evident by altered polysome occupancy of its target transcripts when Ssd1 is not phosphorylated by Cbk1 [6]. To assess ribosome occupancy of exogenous GFP reporters we assayed ribosome density along the Ssd1-regulated *TEF1^5′UTR^-GFP-CTS1^3′UTR^* reporter transcript by polysome profiling, using sucrose gradients to separate bulk mRNPs of varying levels of ribosome content. We determined the relative abundance of *GFP* transcript in monosomes and polysomes by northern blotting mRNA recovered from fractionated sucrose gradients. We compared 1NA-PP1-treated *cbk1-as* cells, where Ssd1 is hyperactive, to 1NA-PP1-treated *cbk1-as ssd1Δ* cells, where Ssd1 is absent. Hyperactivation of Ssd1 did not alter the bulk mRNP polysome profile as measured by optical density at 254 nm (Figure 4A), consistent with prior findings [6]. In cells containing hyperactived Ssd1, abundance of *GFP* mRNA was increased in monosome fractions, (Figure 4B). The housekeeping gene *PGK1*, itself not a target of Ssd1, showed invariant polysome and monosome association when Ssd1 was hyperactived (Figure 4B). Polysome profiling is a qualitative assay subject to inherent variations in density gradient preparation or northern blot analysis; to further quantify our polysome association experiments, we measured the area under the curve (AUC) of the polysome- and monosome-associated regions (determined by bulk mRNP profiles) of both *GFP* and *PGK1* relative mRNA abundance profiles across three replicate experiments (Figure 4B and Figure S5A). Polysome occupancy of *GFP-CTS1^3′UTR^* was significantly reduced (p = 0.04056) in cells where Ssd1 was hyperactivated, while the *PGK1* mRNA was not (Figures S5B and S5C). While statistically significant, monosome association of our GFP reporter in response to Ssd1 hyperactivation was modest, particularly when compared to the shift that occurs with endogenous Ssd1-associated mRNAs [6]. This may be attributable to short length of the *GFP* transcript, which likely reduces overall maximal polysome density.

### 3′UTRs of some Ssd1 targets confer Ssd1-mRNA interaction

**Figure 5:**
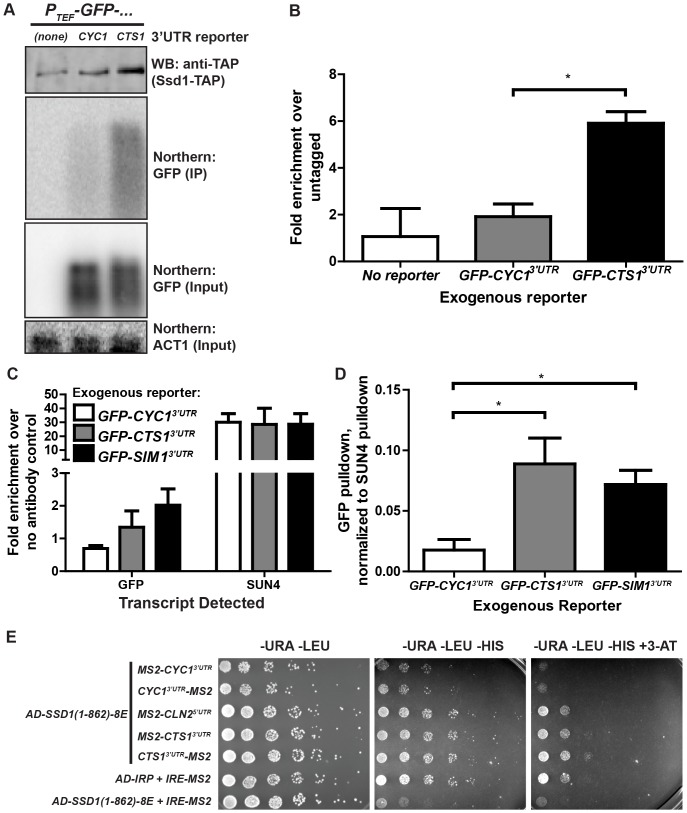
The 3′UTRs of *CTS1* and *SIM1* confer Ssd1 binding to GFP reporters. **(A)** Representative samples of Western and Northern blots show TAP-tagged Ssd1 immunoprecipitates *P_TEF1_-GFP-CTS1^3′UTR^* reporter. In extracts from strains expressing Ssd1-TAP (see WB: anti-TAP), *GFP* mRNA is immunoprecipitated when the *CTS1*, but not the *CYC1*, 3′UTR is present (see Northern: GFP (IP)). In RNA immunoprecipitation input samples, *GFP* mRNA was detected irrespective of its 3′UTR and *ACT1* mRNA was present in all samples. **(B)** The fold enrichment of GFP mRNA immunoprecipitated from *SSD1-TAP* over *SSD1* (untagged) cells was quantified over three experiments. The GFP reporter expressed in the context of the *CTS1* 3′UTR was significantly enriched in SSD1-TAP IP samples compared to reporters bearing the *CYC1* 3′UTR. **(C)** *GFP-CTS1^3′UTR^* and *GFP-SIM1^3′UTR^* reporters, but not the control *GFP-CYC1^3′UTR^* reporter, are immunoprecipitated in *SSD1-TAP* lysates incubated with anti-TAP antibody. Real-time qPCR detection shows variant pull-down efficiency across 3 experiments, as shown by the variance in *SUN4* mRNA immunoprecipitation. **(D)** Three replicate RNA IP experiments were normalized using *SUN4* mRNA IP as a positive control. *GFP-CTS1^3′UTR^* and *GFP-SIM1^3′UTR^* reporters show significant enrichment in RNA IP samples compared to *GFP-CYC1^3′UTR^* reporter. Error bars represent ± SEM and * indicates P-value 0.01 to 0.05 at 95% confidence intervals as calculated by unpaired two-tailed Student's t-test. **(E)** *CTS1* 3′UTR and *CLN2* 5′UTR confer 3-hybrid interaction with Ssd1. In either MS2 aptamer position, the *CYC1* 3′UTR does not mediate a 3-hybrid interaction, while the *CTS1* 3′UTR does mediate a 3-hybrid interaction. Notably, the *CLN2* 5′UTR also mediates a 3-hybrid interaction. AD-IRP co-transformed with *IRE-MS2* serves as a positive control, while AD-Ssd1-(1-862)-8E co-transformed with *IRE-MS2* serves as a negative control.

We sought to determine if UTRs that confer Ssd1-mediated translational repression could physically associate with Ssd1. We performed RNA immunoprecipitation assays to determine if the *CTS1* 3′UTR could mediate immunoprecipitation of *GFP* mRNA with TAP-tagged Ssd1. We found that while *GFP* mRNA produced from either the *GFP-CYC1^3′UTR^* or *GFP-CTS1^3′UTR^* reporters was present in lysates, only the *CTS1* 3′UTR conferred significant co-precipitation with Ssd1-TAP (Figure 5A). Correcting for the amount of Ssd1-TAP immunoprecipitated across two experiments, we found significant enrichment (p = 0.0319) of *GFP* transcript in *SSD1-TAP* compared to *SSD1* cells only when the *CTS1* 3′UTR is present (Figure 5B).

We next tested the *SIM1* 3′UTR's ability to confer Ssd1 association with mRNA, comparing its pull-down with the *CTS1* 3′UTR reporter and the endogenous *SUN4* transcript. We found elevated immunoprecipitation efficiency of *GFP* mRNA when the *CTS1* 3′UTR or *SIM1* 3′UTR is present compared to the *CYC1* 3′UTR reporter, which was not enriched over a no antibody control (Figure 5C). Robust immunoprecipitation of the *SUN4* endogenous transcript, as previously reported [6], revealed variations in overall pull-down efficiency between biological and technical replicates; we thus normalized *GFP* mRNA pull-down to the positive control *SUN4* for each sample in respective experiments. Both the *GFP-CTS1^3′UTR^* and *GFP-SIM1^3′UTR^* reporters showed significant enrichment (*CTS1*: p = 0.0371; *SIM1*: p = 0.0208) in immunoprecipitated samples compared to the *GFP-CYC1^3′UTR^* reporter (Figure 5D). Notably, the enrichment of either the *GFP-CTS1^3′UTR^* or *GFP-SIM1^3′UTR^* reporters were at most 10-fold less than that of the endogenous transcript *SUN4*. This is consistent with a previously observed 10-fold difference between Ssd1's association with *SUN4* and with *UTH1, CTS1* and *SIM1* [6]. Despite these differences in Ssd1 binding efficiency, there was little correlation with the strength of translational control of GFP reporters (Figure 2). Overall, these results show that at least two 3′UTR elements from known Ssd1-bound transcripts can confer Ssd1 binding to an otherwise unassociated transcript.

We used a yeast 3-hybrid system to test the *CTS1* 3′UTR's interaction with Ssd1 in a different way. We found the *CTS1* 3′UTR, but not the *CYC1* 3′UTR or *IRE* (Iron Response Element), mediated 3-hybrid interaction with a truncated form of Ssd1 (1-862) that includes the RNA binding domain [4]. As a positive control, we also confirmed that the Ssd1-associated *CLN2* 5′UTR [22] exhibited a similarly robust interaction (Figure 5E). Interestingly, although Ssd1 interaction with the CTD tail of RNA polymerase II [47] suggests that Ssd1 may load onto its target transcripts co-transcriptionally, the MS2 hybrid RNAs in the 3-hybrid system we employed are transcribed by RNA polymerase III [35].

### The SIM1 3′UTR enhances Ssd1 interaction with the endogenous SIM1 transcript

**Figure 6:**
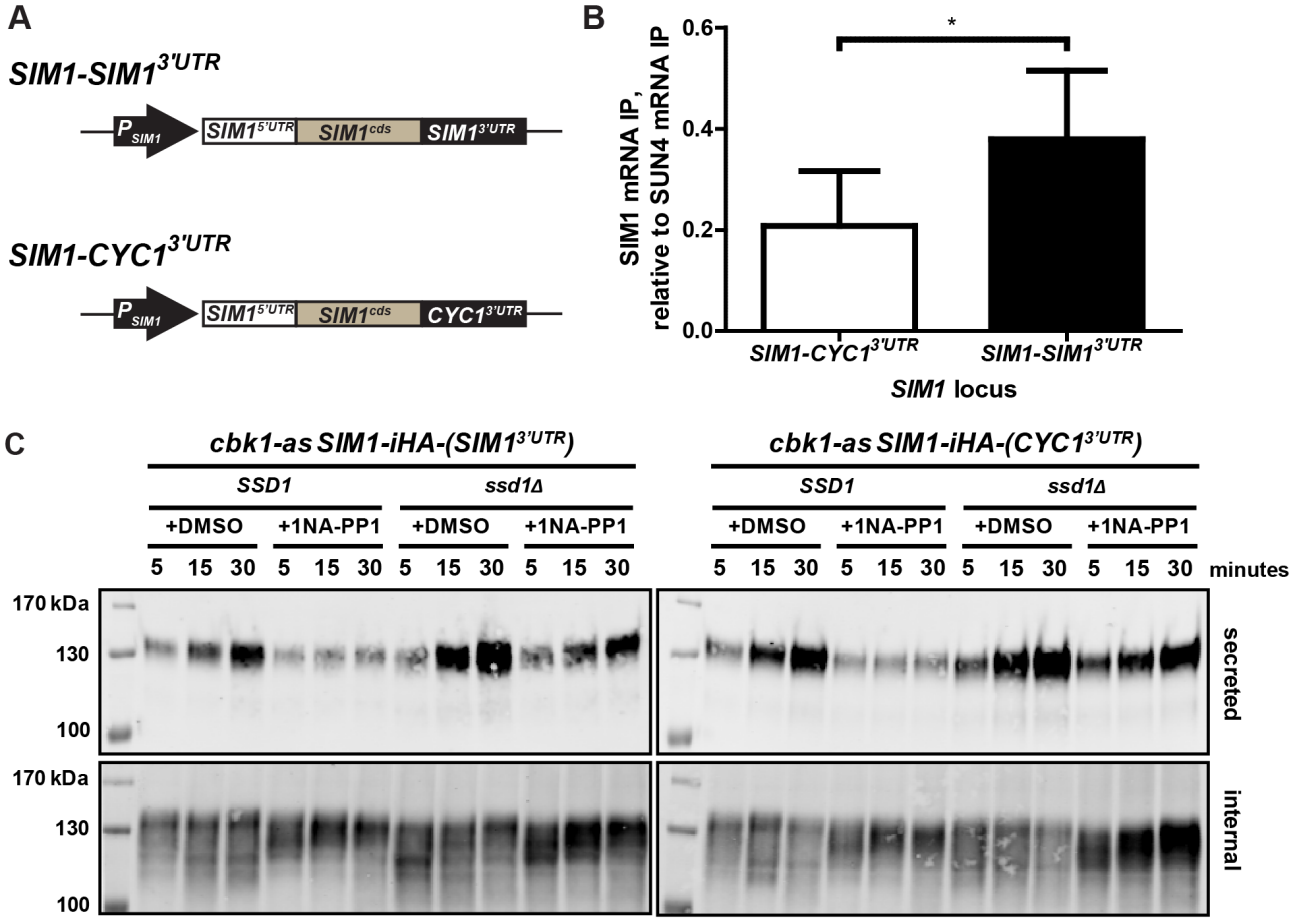
The 3′UTR of *SIM1* is required for efficient Ssd1-SIM1 mRNA interaction but not translational control. **(A)** Diagram of wild-type *SIM1* locus (top) and *SIM1* locus with its endogenous 3′UTR replaced with the *CYC1* 3′UTR (bottom). **(B)** SIM1 mRNA immunoprecipitation of wild-type (*SIM1-SIM1^3′UTR^*) or 3′UTR-ablated (*SIM1-CYC1^3′UTR^*) locus, normalized to the pull-down of the positive control *SUN4*. Three replicate experiments showed a significant difference in the ability of Ssd1 to immunoprecipitate *SIM1* RNA when its endogenous 3′UTR is present compared to when its 3′UTR has been replaced. Ssd1-mRNA immunoprecipitation efficiency was evaluated by comparing *SIM1* enrichment in antibody-treated over antibody-untreated samples and corrected for experimental variability by normalization to the positive control *SUN4*. Error bars represent ± SEM and * indicates P-value 0.01 to 0.05 at 95% confidence intervals as calculated by unpaired two-tailed Student's t-test. **(C)** Ablating the endogenous 3′UTR of the *SIM1* does not disrupt phosphoregulated Ssd1 translational control over Sim1. Western blotting against secreted and internal Sim1 was performed as described in Materials in Methods, from cells harvested at 5, 15 and 30 minutes following media replenishment. In both wild-type (left panels) and 3′UTR-ablated *SIM1* (right panels), secreted Sim1 levels are depleted when Ssd1 is hyperactivated (see *cbk1-as SSD1* +1NA-PP1). Translational repression of Sim1 expressed from both loci is dependent on the presence of Ssd1 (see *cbk1-as ssd1Δ +*1NA-PP1) 1NA-PP1 treatment (see +DMSO lanes).

Having found that 3′UTR elements are sufficient for Ssd1-mediated translational regulation of a GFP reporter, we asked if the 3′UTR of an endogenous Ssd1 target transcript is necessary for Ssd1 association with target mRNAs. We replaced the 3′UTR of the Ssd1 target transcript *SIM1* at its endogenous locus with the *CYC1* 3′UTR (Figure 6A) and compared Ssd1 immunoprecipitation of this chimeric *SIM1-CYC1* ^3′UTR^ transcript and the native *SIM1* transcript. We found that Ssd1 immunoprecipitation of *SIM1-CYC1* ^3′UTR^ was significantly reduced (p = 0.0422), about two-fold, relative to the endogenous *SIM1* transcript (Figure 6B). This difference in precipitation was not a result of variations in the pull-down of Ssd1 between the two test strains (Figure S6), demonstrating that replacing the *SIM1* 3′UTR with an otherwise unbound 3′UTR reduces Ssd1 association with the *SIM1* message.

### Ssd1 exerts translational control over SIM1 through redundant means

Since the *SIM1* 3′UTR promotes Ssd1 association with the *SIM1* mRNA and is sufficient for Ssd1-mediated translational control of a *GFP-SIM1^3′UTR^* construct, we asked if this 3′UTR is necessary for Ssd1's translational repression of the *SIM1* mRNA. The Sim1 protein, like many encoded by Ssd1 target mRNAs, is a secreted cell wall associated protein [6]; these are generally long-lived, complicating measurement of translational suppression. We therefore analyzed the levels of both cell-associated Sim1 and the fraction of Sim1 secreted from cells into growth medium. For the experiments shown in Figure 6, we used *cbk1-as* cells with either *ssd1Δ* or the wild type *SSD1* allele, and expressing either the endogenous *SIM1* mRNA or *SIM1-CYC1^3′UTR^*. We first treated these cells with either DMSO or 1NA-PP1 for about an hour, and then washed them into fresh medium with either DMSO or 1NA-PP1 and took samples of cells and cell-free growth medium at indicated times. When we inhibited cbk1-as in cells containing *SSD1* and wild-type *SIM1* the amount of Sim1 secreted into the media was greatly reduced relative to control DMSO treatment (Figure 6C, left). As expected for a protein with slow degradation, we did not see extensive depletion of cell-associated Sim1 upon Cbk1-as inhibition in *SSD1* cells. We found *SIM1-CYC1^3′UTR^* behaved essentially identically to the endogenous *SIM1* mRNA in all assays (Figure 6C, right). These experiments indicate that hyperactivation of Ssd1 represses translation of Sim1, and that the *SIM1* 3′UTR is not necessary for this effect.

Consistent with absence of translational repression, we saw no significant reduction in the amount of secreted Sim1 in 1NA-PP1-treated *cbk1-as ssd1Δ* cells with either the endogenous *SIM1* gene or the *SIM1-CYC1^3′UTR^* chimera. Under these conditions, the amount of cell-associated Sim1 was increased. We found that *SIM1* mRNA levels were significantly elevated in 1NA-PP1-treated *cbk1-as ssd1Δ* cells (Figure S7), which we infer reflects increased *SIM1* transcription and results in a corresponding increase in the amount of cell-associated Sim1.

## Discussion and Conclusions

**Figure 7:**
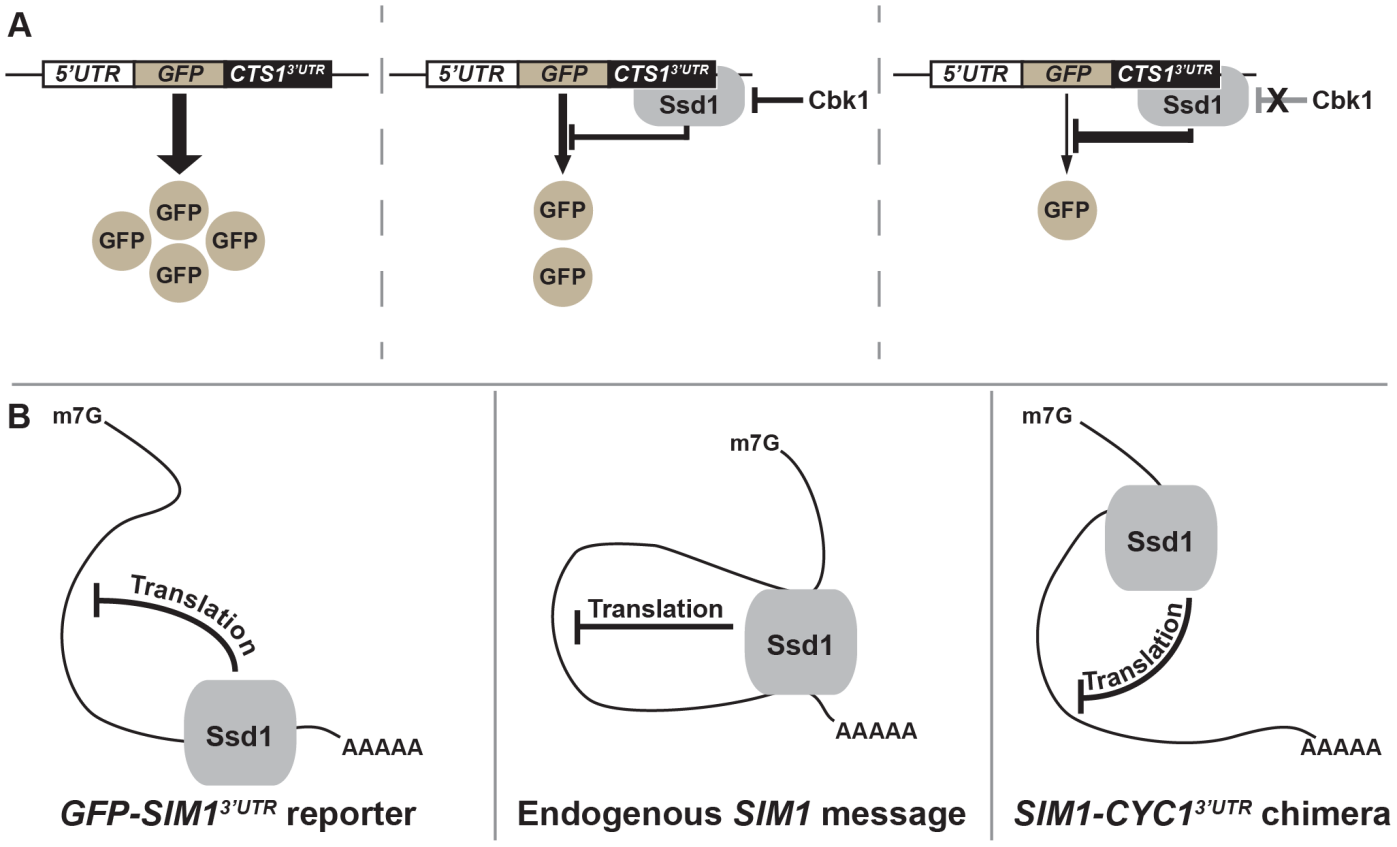
Visual representation of Ssd1-mediated translational control of GFP reporters and multivalent interaction with *SIM1*. (A) Depiction of our observed results in this study: left, a reporter mRNA bearing an Ssd1-bound UTR produces GFP in an unregulated manner; center, the presence of Ssd1 subjects this reporter mRNA to Ssd1 binding and translational control, resulting in reduced expression; right, Cbk1 inhibition hyperactivates the UTR-bound Ssd1 and represses the translation of the protein encoded by the Ssd1-associated mRNA. (B) Ssd1 may interact both directly with the *SIM1* transcript's 3′UTR and its 5′UTR in a ‘closed-loop’ mRNA configuration. This is a depiction of our observation that ablating the 3′UTR of the Ssd1-bound message *SIM1* reduces immunoprecipitation efficiency but does not disrupt the ability of Ssd1 to confer translational repression. Left, a reporter expressing only the 3′UTR of an Ssd1-bound messages confers direct binding, permitting translational repression; center, the endogenous *SIM1* transcript containing both native 5′ and 3′UTRs confers Ssd1 binding through interaction with a 5′UTR-bound RNA binding protein and through direct RNA binding at the 3′UTR; right, the endogenous *SIM1* transcript lacking its native 3′UTR is no longer receptive to direct Ssd1-mRNA interaction at its 3′UTR but still permits Ssd1 binding and translational repression at its 5′UTR. We emphasize that this panel describes our results for the *SIM1* transcript and may not be a general mode of Ssd1-mRNA interaction. Note that all depicted Ssd1-mRNA interaction may not be direct.

Figure 7 presents a graphical summary of our analysis of *CTS1* and *SIM1 UTRs* in Ssd1's binding and translational control of these mRNAs. While not a general model for Ssd1-mRNA interaction and translational repression, our results indicate that the *CTS1* and *SIM1* 3′UTRs are sufficient for Ssd1-mediated translational control and binding in heterologous contexts. Ssd1-mediated translational repression of these constructs is particularly strong when the Ndr/LATS kinase Cbk1 is inhibited, consistent with this kinase's direct negative regulation of Ssd1[6]. Intriguingly, the *SIM1* 3′UTR helps confer robust Ssd1 mRNA binding but is not essential for Ssd1 translational control of the SIM1 mRNA. We therefore suggest that information in both 5′ and 3′ UTRs can play a role in *SIM1* repression. Consistent with this, we find that Ssd1 can exert translational control over the *UTH1* mRNA through either its 5′ or 3′UTR (Figure 2D).

As noted, the SEE motif is clearly enriched in the 5′UTRs of some known Ssd1 target transcripts [5], [22]. There is no direct evidence that it binds Ssd1 or directs translational control, and not all Ssd1 target transcripts contain the SEE in 5′UTR regions [5], [6]. We find that the SEE-containing 5′UTR of *CTS1* does not mediate Ssd1-mediated translational regulation of reporter constructs, while other mRNA regions that do not contain this motif can do this. It is notable that the *UTH1* 5′UTR, which contains four SEE motifs, confers translational control. Thus, it remains possible that the SEE promotes Ssd1-mediated translational control in some contexts. However, the SEE itself appears to be neither necessary nor sufficient to for Ssd1 translational control of mRNAs. Overall, our findings suggest that multiple signals can direct Ssd1 to target transcripts, and that the SEE is one of several mechanisms that promote Ssd1 association with a target mRNP.

Our analysis of Ssd1's association with specific transcripts [6], [22] does not discriminate between direct interaction of Ssd1 with mRNA and indirect interaction through another RNA binding protein, several of which are known to associate with Ssd1 [6]. Thus, Ssd1 may influence translation by binding to another protein that interacts with specific mRNAs. Ssd1 association with the *CLN2 5′UTR* and *CTS1* 3′UTR by yeast three-hybrid is especially notable, as the hybrid RNAs used in this system are transcribe from an RNA polymerase III promoter. There is strong evidence that Ssd1 associates with the Ser2,5P CTD tail of RNA polymerase II [47], a hallmark of transcriptional elongation, and previous studies have suggested co-transcriptional loading of RNA binding proteins [5]. Our three-hybrid data suggest that co-transcriptional loading of RNA polymerase II-associated Ssd1 onto an mRNP is not the exclusive mechanism by which Ssd1 binds its target transcripts.

Taken together with previously characterized interactions, our findings indicate that that Ssd1 may be present in a closed loop mRNP configuration that permits multiple points of Ssd1-mRNP contact. We have shown that *CTS1* and *SIM1* 3′UTRs confer Ssd1 translational control, as can both the 5′ and 3′ UTRs of the *UTH1* mRNA; Ssd1 associates with the *CLN2* 5′UTR [22] and the polyA-binding protein Pab1 [23]. Thus, *cis-*elements or proteins bound in both 5′ and 3′UTRs may work together, and possibly redundantly, to create a context amenable to Ssd1 association and function. Consistent with redundancy of Ssd1 translational control, the *SIM1* 3′UTR is sufficient for Ssd1-mediated translational control, but is not essential in the endogenous context of *SIM1* (Figure 7B). Intriguingly, analysis of Ssd1 binding to the *CLN2* 5′UTR indicates that this association stabilizes Cln2 expression [22], distinct from Ssd1 repression of the translation of bound transcripts [6]. Thus, it is possible that Ssd1 exerts variable effects over an mRNA's behavior depending on context and the complement of RNA binding proteins present on the transcript.

Our results show that Ssd1 can tune expression of proteins through association with UTRs in their mRNAs. In at least some cases, this Ssd1 association allows the Ndr/LATS kinase Cbk1 to control the mRNA's translation. Ssd1-mediated translational control was not universal for all of the UTRs we tested. This could indicate either a limitation of reporters in studying RBP-mediated translational control, or that Ssd1 regulons present in some UTRs (such as *SCW10* or *TOS1*) may not function in a heterologous context, possibly due to a changed complement of RBPs or mRNA secondary structure. *SUN4* presents an intriguing case. We found that neither its 5′ nor 3′ UTRs are sufficient to confer Ssd1-mediated translational control, and perhaps the *SUN4* 5′ and 3′ UTRs must flank the appropriate ORF for Ssd1 translational repression. Interestingly, the SEE motif is present in the *SUN4* ORF, but not in the *SUN4* UTRs. Other Ssd1-bound transcripts not tested here may also have functionally distinct mechanisms that promote Ssd1 association. These sites may serve complementary roles in shaping the spatiotemporal expression of proteins encoded by Ssd1 target transcripts, influencing translation, RNA localization and stability in diverse ways. This would allow the Ssd1 system to influence a wide range of processes involved in cell wall maintenance and robustness.

## Supporting Information

Figure S1 **GFP bearing the Ssd1-bound transcript** ***CTS1's*** **3′UTR is differentially expressed depending on Ssd1 genotype.** GFP fused to the *CTS1* 3′UTR shows significantly depressed expression in *SSD1* cells, determined by: **(A)**, the ratio of GFP MFI of [*P_TEF1_-GFP-CYC1^3′UTR^*] or [*P_TEF1_-GFP-CTS1^3′UTR^*] in *ssd1Δ* over *SSD1* cells and **(B)**, the ratio of [*P_TEF1_-GFP-CTS1^3′UTR^*] to [*P_TEF1_-GFP-CYC1^3′UTR^*] GFP MFI in *ssd1Δ* or *SSD1* cells, determined by flow cytometry. **(C)** GFP transcription of reporters is not significantly different as determined by real-time qPCR. Data presented in (A) through (C) represent at least three independent trials. Error bars represent ± SEM, *** indicates P-value<0.001, ‘ns’ indicates P-value>0.05 at 95% confidence intervals as calculated by unpaired two-tailed Student's t-test.
(TIF)

Figure S2 **UTRs from further Ssd1 targets do not confer translational control.** We expressed GFP reporters with the indicated 3′ or 5′UTR in *SSD1* and *ssd1Δ* cells and evaluated their expression by measuring MFI on a flow cytometer as described in Materials in Methods. **(A)** The 3′UTRs from the Ssd1-associated messages *SUN4, TOS1* and *SCW10* do not confer significant Ssd1-dependent variations in expression compared to the control reporter bearing the *CYC1* 3′UTR. **(B)** The 5′UTRs from the Ssd1-associated messages *SUN4* and *CTS1* do not confer significant Ssd1-dependent variations in expression compared to control reporters bearing either the *TEF1* or *ADH1* 5′UTR. The expression of these constructs is nearly identical in *SSD1* and *ssd1Δ* cells. Data represent three independent trials. Error bars represent ± SEM. No P-values calculated between control (gray bars) and test (black bars) constructs were significant (P-value>0.05 at 95% confidence intervals as calculated by unpaired two-tailed Student's t-test.)
(TIF)

Figure S3 **(A)** Stable GFP-*CTS1^3′UTR^* or **(B)** -*SIM1^3′UTR^* reporters do not respond to 1 hour 1NA-PP1 treatment (compare *cbk1-as* +DMSO and *cbk1-as* +1NA-PP1) as measured by GFP MFI. Expression in *cbk1-as SSD1* is significantly different to expression in *CBK1 ssd1Δ* cells, showing that the *cbk1-as* allele retains kinase activity. GFP perdurance likely masks changes in GFP translation in response to 1NA-PP1 treatment, necessitating the use of destabilized GFP^PEST^. In (A) and (B), fluorescence data were corrected for variations in cell size apparent in forward scatter (FSC) measurements as described in Materials and Methods. Error bars represent ± SEM, ** indicates P-value of 0.001 to 0.01, * indicates P-value 0.01 to 0.05, and ‘ns’ indicates P-value>0.05 at 95% confidence intervals as calculated by unpaired two-tailed Student's t-test.
(TIF)

Figure S4 **Destabilized GFP-CTS1^3′UTR^ reporters respond to Cbk1 inhibition and are further repressed under growth in ethanol.** **(A)** Western blotting against GFP confirms destabilized GFP (GFP-Cln2^PEST^) bearing the *CTS1* 3′UTR expression is responsive to Ssd1 phosphorylation state and is depleted on Cbk1 inhibition when expressed from either *ADH1 or TEF1* promoters, while steady-state levels of the housekeeping gene Pgk1 are unaffected. **(B)** Repression of destabilized GFP reporter (uGFP) expression under Cbk1 inhibition depends on the presence of Ssd1 or an Ssd1-regulated 3′UTR. We report the relative %GFP positive at each time point t>1 h as a fold change relative to the %GFP positive population at t = 0 h. Flow cytometry was performed on cells fixed at one hour intervals as described in Materials in Methods. **(C)** Reporter expression under growth in 5% ethanol, a condition where Ssd1 function is critical, was examined by flow cytometry as described in Materials and Methods. Histograms depicting the GFP fluorescence of *CBK1 SSD1* or *cbk1-as SSD1* cells expressing either the Ssd1-bound destabilized reporter (*uGFP-CTS1^3′UTR^*) or unbound reporter (*uGFP-CYC1^3′UTR^*), grown in YPD rich media supplemented to 5% or 0% (v/v) final ethanol concentration reveal strong suppression of GFP expression in ethanol-exposed *cbk1-as* cells expressing the bound *CTS1* 3′UTR reporter, but not in *CBK1* cells or when an unbound *CYC1* 3′UTR is expressed.
(TIF)

Figure S5 **Ssd1-regulated expression of GFP reporter is due to changes in transcript ribosomal occupancy.** Polysome profiling of RNA extracts followed by Northern blot analysis of RNA fractions from across the polysome gradient were used to analyze ribosomal occupancy of the *P_TEF1_-GFP-CTS1^3′UTR^* reporter in 1NA-PP1-treated *cbk1-as SSD1* and *cbk1-as ssd1Δ cells*. Experiments were performed as described in Figure 4; here, we show two additional replicates. **(A)** Relative mRNA abundance traces from Northern blots of two replicate polysome profiling experiments (Trial 2, top and Trial 3, bottom). Highlighted gray regions indicate the mRNA fractions associated with polysomes, determined from total A_254_ measurements of fractionated sucrose gradients. *GFP* mRNA is enriched in monosomes in the absence of Ssd1, while *PGK1* mRNA polysome association changes minimally. **(B)** Three replicate *GFP* and *PGK1* ribosomal occupancy maps were analyzed by calculating the total area under the curve (AUC) and determining the fraction of that area encompassed by the polysome-associated region (gray box). We saw a significant difference in *GFP-CTS1^3′UTR^* polysome association, but not for *PGK1*. **(C)** Data tables for *GFP* and *PGK1* ribosome AUC calculations show the percent encompassed by the polysome region of each trial, the mean percentage of each transcript in polysome regions, and P-value. Error bars represent ± SEM, * indicates P-value 0.01 to 0.05, and ‘ns’ indicates P-value>0.05 at 95% confidence intervals as calculated by unpaired two-tailed Student's t-test.
(TIF)

Figure S6 **Immunoprecipitation of Ssd1 is similar is not affected by 3′UTR identify at the** ***SIM1*** **locus.** Samples representing 0.05% (v/v) of the volume at each indicated experimental stage were removed for SDS-PAGE analysis by Western blotting with anti-TAP. Ssd1 protein is similarly immunoprecipitated in samples expressing the *SIM1* locus with either the *SIM1* or *CYC1* 3′UTR.
(TIF)

Figure S7 ***SIM1*** **transcription increases in 1NA-PP1-treated** ***cbk1-as ssd1Δ*** **cells.** We collected mRNA from cells used in the assay for secreted Sim1 (Figure 6C) and measured *SIM1* message abundance by quantitative RT-PCR. As noted in our discussion of Figure 6C, we saw increased cell-associated Sim1 protein in *cbk1-as ssd1Δ* cells treated with 10 µM 1NA-PP1 for 1 hour. *SIM1* message levels were significantly elevated in 1NA-PP1-treated *cbk1-as ssd1Δ* cells compared to *cbk1-as SSD1* cells with the same treatment, and significantly elevated compared to DMSO-treated *cbk1-as ssd1Δ* cells. We saw no significant difference in *SIM1* transcript abundance between *cbk1-as SSD1* and *cbk1-as ssd1Δ* cells treated with DMSO. Data shown are the result of four independent trials, each of which included three technical triplicates. Error bars represent ± SEM, * indicates P-value 0.01 to 0.05, and ‘ns’ indicates P-value>0.05 at 95% confidence intervals as calculated by unpaired two-tailed Student's t-test.
(TIF)
