# Supplementary figures and images for: Cell Morphogenesis Proteins Are Translationally Controlled through UTRs by the Ndr/LATS Target Ssd1
Source: PLoS One. 2014 Jan 21;9(1):e85212. doi: 10.1371/journal.pone.0085212 (PMC3897418; doi:10.1371/journal.pone.0085212)

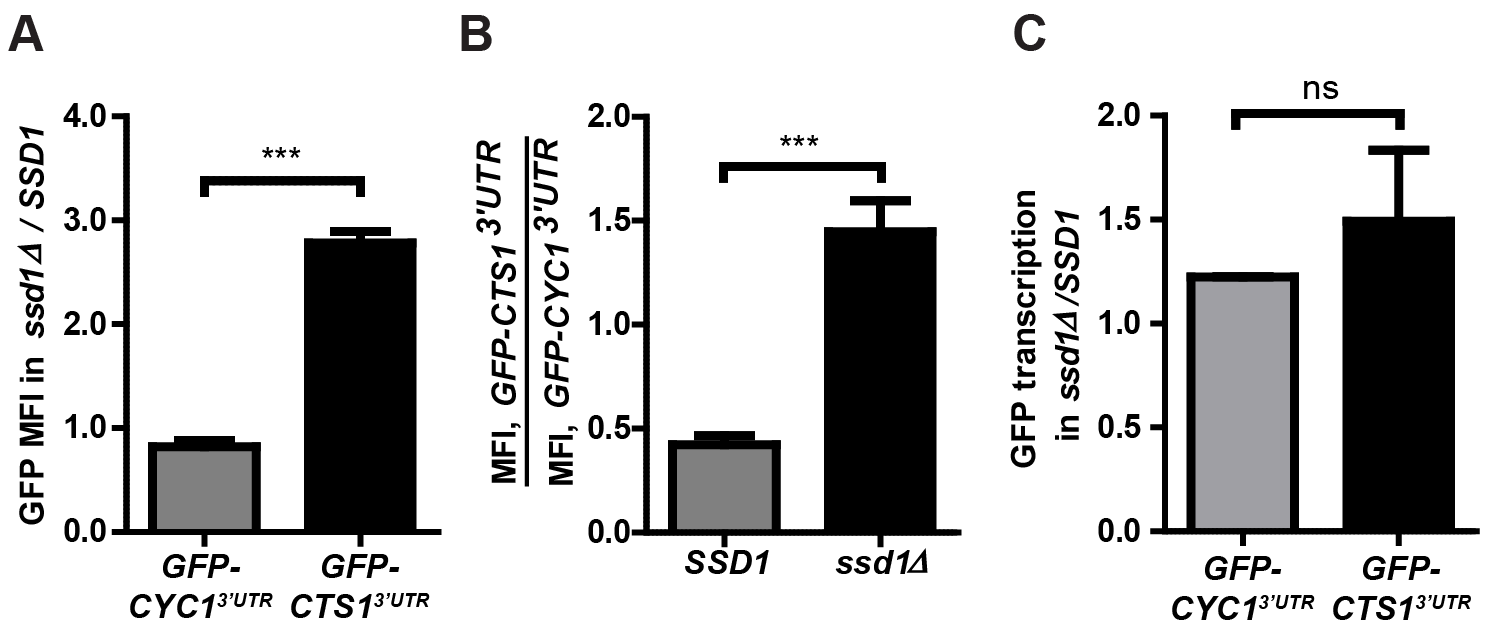

Supplement: Figure S1 — GFP bearing the Ssd1-bound transcript CTS1's 3′UTR is differentially expressed depending on Ssd1 genotype. GFP fused to the CTS1 3′UTR shows significantly depressed expression in SSD1 cells, determined by: (A), the ratio of GFP MFI of [PTEF1-GFP-CYC13′UTR] or [PTEF1-GFP-CTS13′UTR] in ssd1Δ over SSD1 cells and (B), the ratio of [PTEF1-GFP-CTS13′UTR] to [PTEF1-GFP-CYC13′UTR] GFP MFI in ssd1Δ or SSD1 cells, determined by flow cytometry. (C) GFP transcription of reporters is not significantly different as determined by real-time qPCR. Data presented in (A) through (C) represent at least three independent trials. Error bars represent ± SEM, *** indicates P-value<0.001, ‘ns’ indicates P-value>0.05 at 95% confidence intervals as calculated by unpaired two-tailed Student's t-test. (TIF) [file pone.0085212.s001.tif]

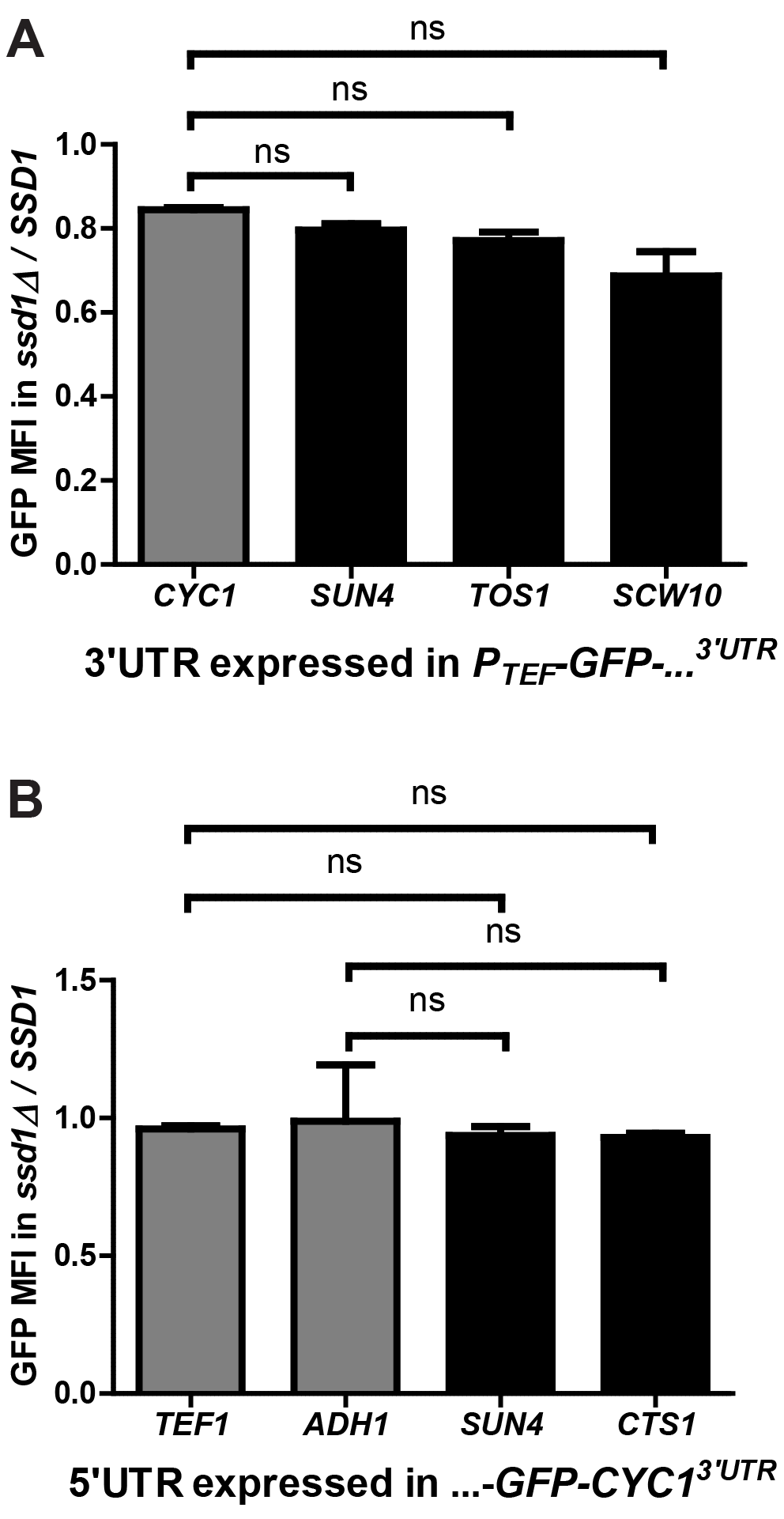

Supplement: Figure S2 — UTRs from further Ssd1 targets do not confer translational control. We expressed GFP reporters with the indicated 3′ or 5′UTR in SSD1 and ssd1Δ cells and evaluated their expression by measuring MFI on a flow cytometer as described in Materials in Methods. (A) The 3′UTRs from the Ssd1-associated messages SUN4, TOS1 and SCW10 do not confer significant Ssd1-dependent variations in expression compared to the control reporter bearing the CYC1 3′UTR. (B) The 5′UTRs from the Ssd1-associated messages SUN4 and CTS1 do not confer significant Ssd1-dependent variations in expression compared to control reporters bearing either the TEF1 or ADH1 5′UTR. The expression of these constructs is nearly identical in SSD1 and ssd1Δ cells. Data represent three independent trials. Error bars represent ± SEM. No P-values calculated between control (gray bars) and test (black bars) constructs were significant (P-value>0.05 at 95% confidence intervals as calculated by unpaired two-tailed Student's t-test.) (TIF) [file pone.0085212.s002.tif]

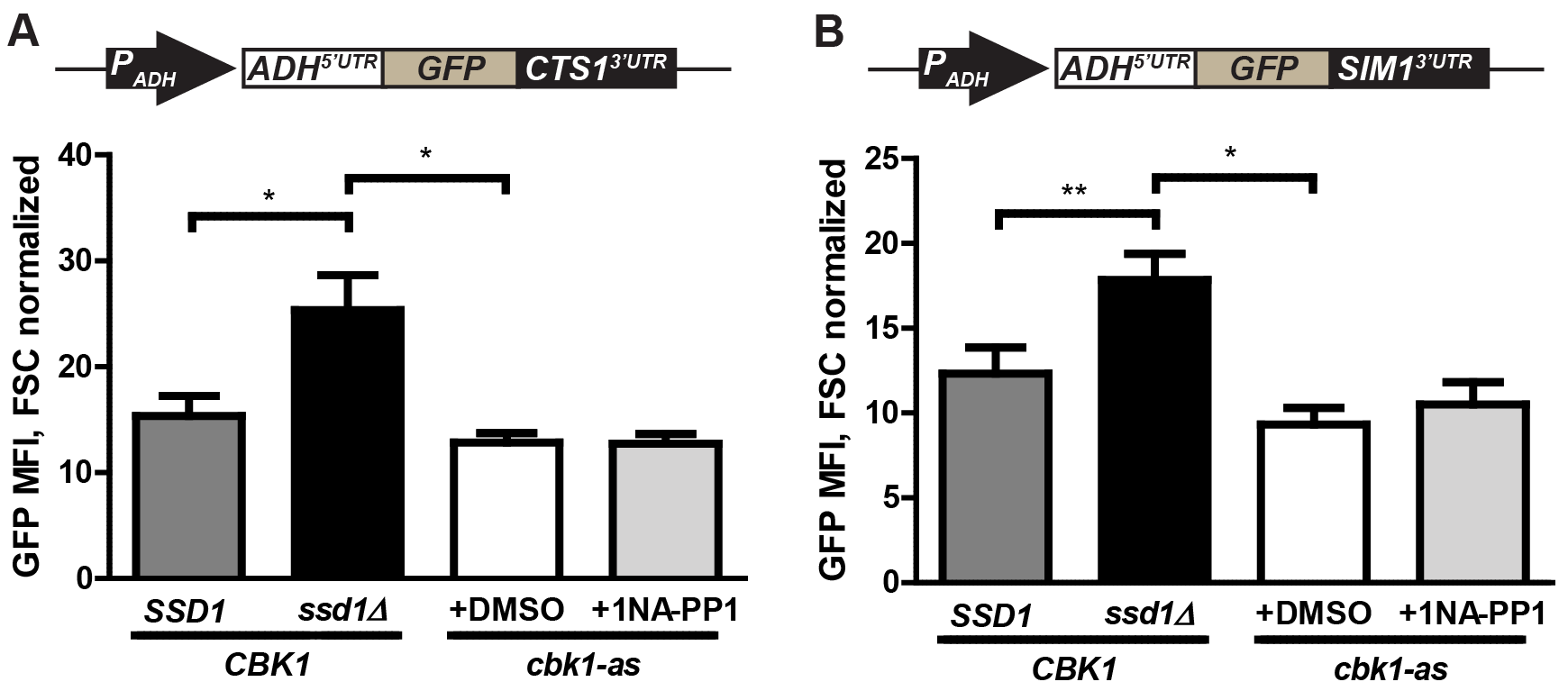

Supplement: Figure S3 — (A) Stable GFP-CTS13′UTR or (B) -SIM13′UTR reporters do not respond to 1 hour 1NA-PP1 treatment (compare cbk1-as +DMSO and cbk1-as +1NA-PP1) as measured by GFP MFI. Expression in cbk1-as SSD1 is significantly different to expression in CBK1 ssd1Δ cells, showing that the cbk1-as allele retains kinase activity. GFP perdurance likely masks changes in GFP translation in response to 1NA-PP1 treatment, necessitating the use of destabilized GFPPEST. In (A) and (B), fluorescence data were corrected for variations in cell size apparent in forward scatter (FSC) measurements as described in Materials and Methods. Error bars represent ± SEM, ** indicates P-value of 0.001 to 0.01, * indicates P-value 0.01 to 0.05, and ‘ns’ indicates P-value>0.05 at 95% confidence intervals as calculated by unpaired two-tailed Student's t-test. (TIF) [file pone.0085212.s003.tif]

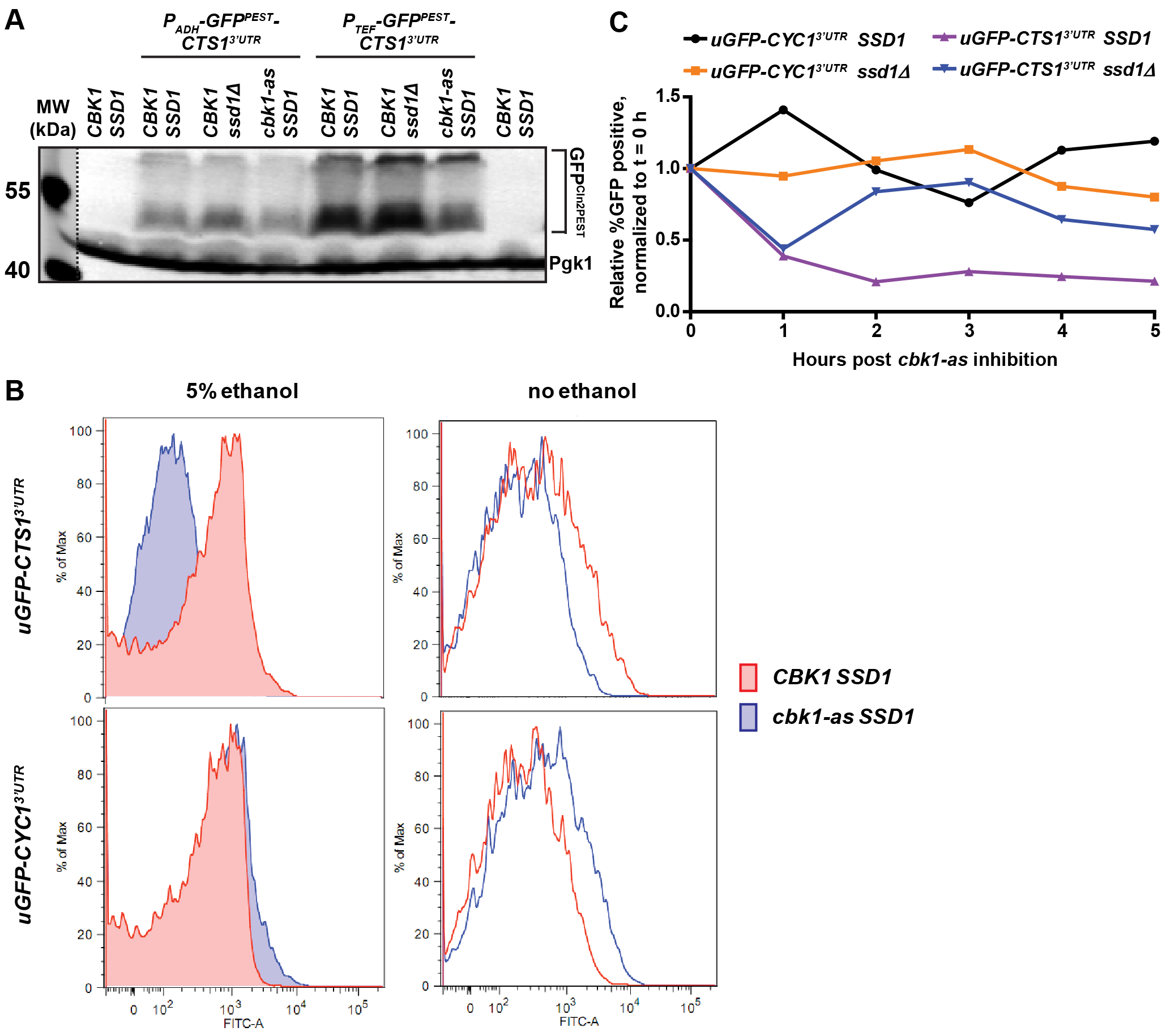

Supplement: Figure S4 — Destabilized GFP-CTS13′UTR reporters respond to Cbk1 inhibition and are further repressed under growth in ethanol. (A) Western blotting against GFP confirms destabilized GFP (GFP-Cln2PEST) bearing the CTS1 3′UTR expression is responsive to Ssd1 phosphorylation state and is depleted on Cbk1 inhibition when expressed from either ADH1 or TEF1 promoters, while steady-state levels of the housekeeping gene Pgk1 are unaffected. (B) Repression of destabilized GFP reporter (uGFP) expression under Cbk1 inhibition depends on the presence of Ssd1 or an Ssd1-regulated 3′UTR. We report the relative %GFP positive at each time point t>1 h as a fold change relative to the %GFP positive population at t = 0 h. Flow cytometry was performed on cells fixed at one hour intervals as described in Materials in Methods. (C) Reporter expression under growth in 5% ethanol, a condition where Ssd1 function is critical, was examined by flow cytometry as described in Materials and Methods. Histograms depicting the GFP fluorescence of CBK1 SSD1 or cbk1-as SSD1 cells expressing either the Ssd1-bound destabilized reporter (uGFP-CTS13′UTR) or unbound reporter (uGFP-CYC13′UTR), grown in YPD rich media supplemented to 5% or 0% (v/v) final ethanol concentration reveal strong suppression of GFP expression in ethanol-exposed cbk1-as cells expressing the bound CTS1 3′UTR reporter, but not in CBK1 cells or when an unbound CYC1 3′UTR is expressed. (TIF) [file pone.0085212.s004.tif]

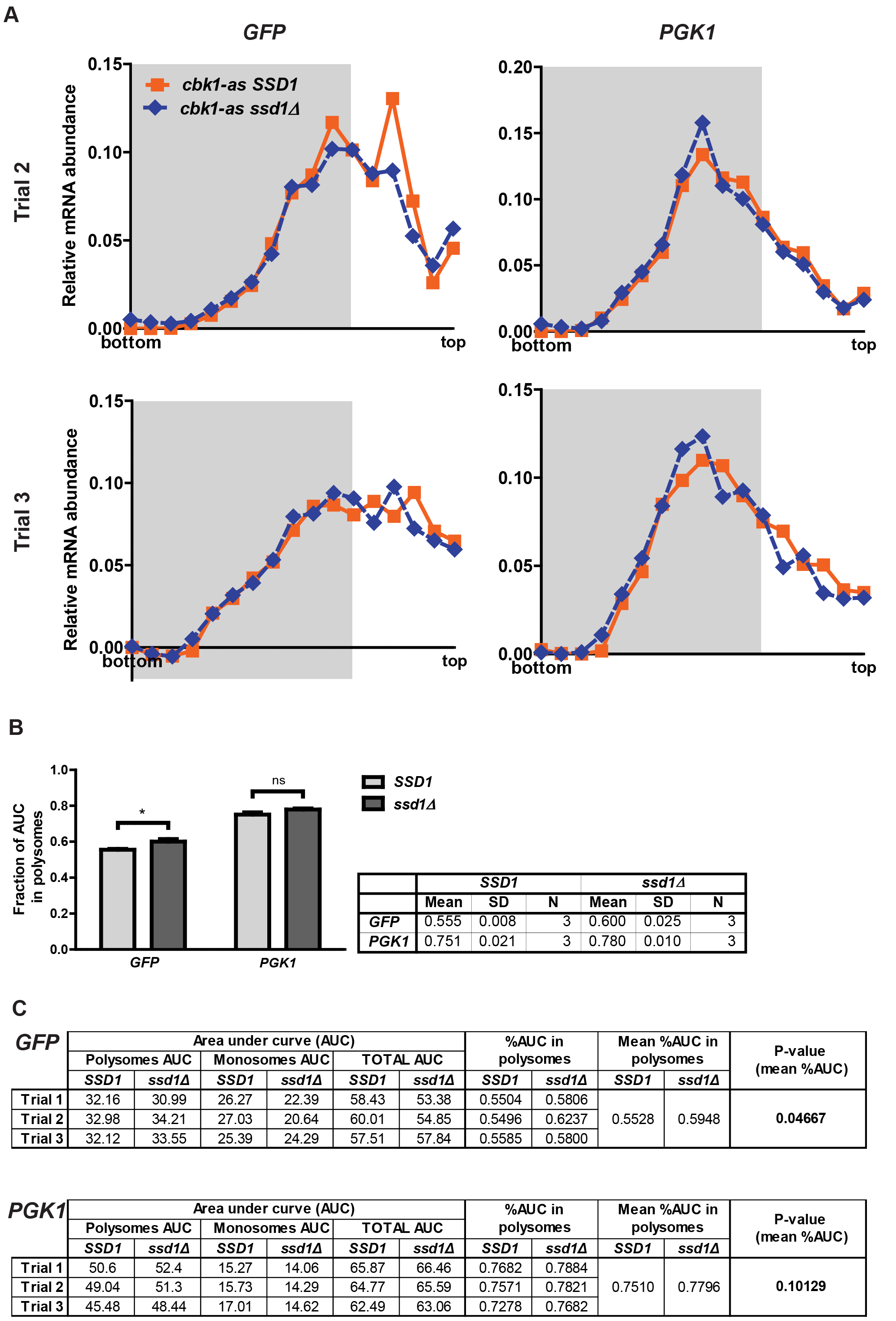

Supplement: Figure S5 — Ssd1-regulated expression of GFP reporter is due to changes in transcript ribosomal occupancy. Polysome profiling of RNA extracts followed by Northern blot analysis of RNA fractions from across the polysome gradient were used to analyze ribosomal occupancy of the PTEF1-GFP-CTS13′UTR reporter in 1NA-PP1-treated cbk1-as SSD1 and cbk1-as ssd1Δ cells. Experiments were performed as described in Figure 4; here, we show two additional replicates. (A) Relative mRNA abundance traces from Northern blots of two replicate polysome profiling experiments (Trial 2, top and Trial 3, bottom). Highlighted gray regions indicate the mRNA fractions associated with polysomes, determined from total A254 measurements of fractionated sucrose gradients. GFP mRNA is enriched in monosomes in the absence of Ssd1, while PGK1 mRNA polysome association changes minimally. (B) Three replicate GFP and PGK1 ribosomal occupancy maps were analyzed by calculating the total area under the curve (AUC) and determining the fraction of that area encompassed by the polysome-associated region (gray box). We saw a significant difference in GFP-CTS13′UTR polysome association, but not for PGK1. (C) Data tables for GFP and PGK1 ribosome AUC calculations show the percent encompassed by the polysome region of each trial, the mean percentage of each transcript in polysome regions, and P-value. Error bars represent ± SEM, * indicates P-value 0.01 to 0.05, and ‘ns’ indicates P-value>0.05 at 95% confidence intervals as calculated by unpaired two-tailed Student's t-test. (TIF) [file pone.0085212.s005.tif]

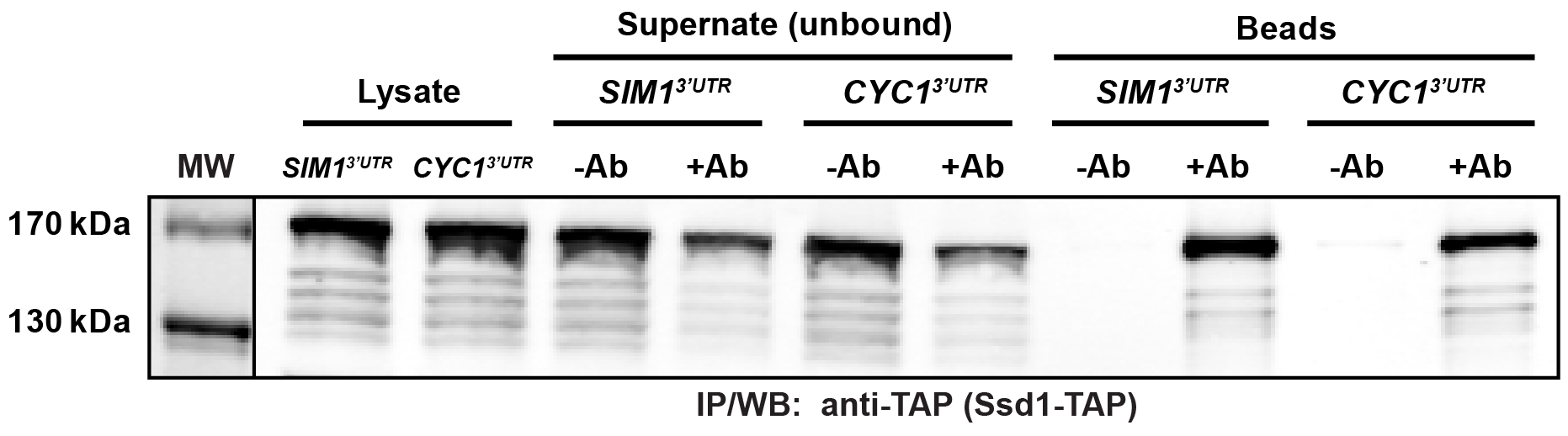

Supplement: Figure S6 — Immunoprecipitation of Ssd1 is similar is not affected by 3′UTR identify at the SIM1 locus. Samples representing 0.05% (v/v) of the volume at each indicated experimental stage were removed for SDS-PAGE analysis by Western blotting with anti-TAP. Ssd1 protein is similarly immunoprecipitated in samples expressing the SIM1 locus with either the SIM1 or CYC1 3′UTR. (TIF) [file pone.0085212.s006.tif]

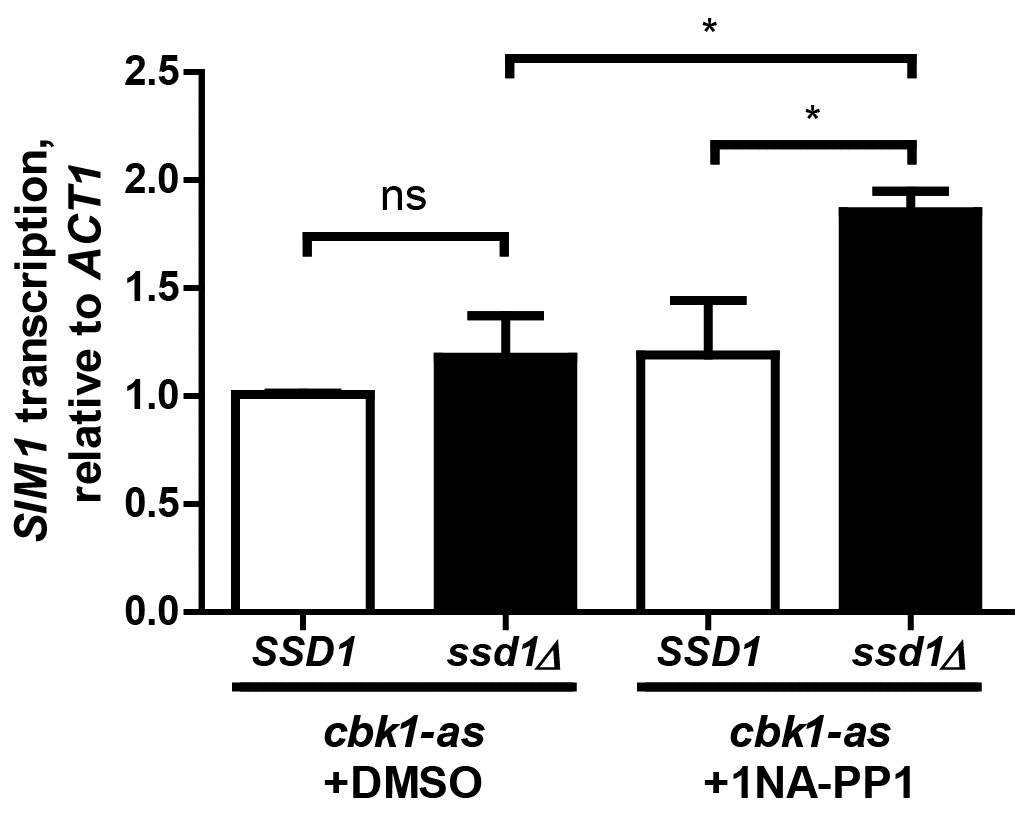

Supplement: Figure S7 — SIM1 transcription increases in 1NA-PP1-treated cbk1-as ssd1Δ cells. We collected mRNA from cells used in the assay for secreted Sim1 (Figure 6C) and measured SIM1 message abundance by quantitative RT-PCR. As noted in our discussion of Figure 6C, we saw increased cell-associated Sim1 protein in cbk1-as ssd1Δ cells treated with 10 µM 1NA-PP1 for 1 hour. SIM1 message levels were significantly elevated in 1NA-PP1-treated cbk1-as ssd1Δ cells compared to cbk1-as SSD1 cells with the same treatment, and significantly elevated compared to DMSO-treated cbk1-as ssd1Δ cells. We saw no significant difference in SIM1 transcript abundance between cbk1-as SSD1 and cbk1-as ssd1Δ cells treated with DMSO. Data shown are the result of four independent trials, each of which included three technical triplicates. Error bars represent ± SEM, * indicates P-value 0.01 to 0.05, and ‘ns’ indicates P-value>0.05 at 95% confidence intervals as calculated by unpaired two-tailed Student's t-test. (TIF) [file pone.0085212.s007.tif]
